# The Cyclin-Dependent Kinase 8 Inhibitor E966-0530-45418 Attenuates Pulmonary Fibrosis *In Vitro* and* In Vivo*

**DOI:** 10.7150/ijbs.105826

**Published:** 2025-01-01

**Authors:** Ching-Hsuan Chou, Wei-Jan Huang, Kai-Cheng Hsu, Jui-Yi Hsu, Tony Eight Lin, Chia-Ron Yang

**Affiliations:** 1 School of Pharmacy, College of Medicine, National Taiwan University, Taipei, Taiwan.; 2 School of Pharmacy, Taipei Medical University, Taipei, Taiwan.; 3 Graduate Institute of Pharmacognosy, College of Pharmacy, Taipei Medical University, Taipei, Taiwan.; 4 Graduate Institute of Cancer Biology and Drug Discovery, College of Medical Science and Technology, Taipei Medical University, Taipei, Taiwan.; 5 Ph.D. Program for Cancer Molecular Biology and Drug Discovery, College of Medical Science and Technology, Taipei Medical University, Taipei, Taiwan.

**Keywords:** pulmonary fibrosis, cyclin-dependent kinase 8, TGFβ1/Smad signaling, transcriptional regulation, drug discovery

## Abstract

Pulmonary fibrosis (PF) is a high-mortality lung disease with limited treatment options, highlighting the need for new therapies. Cyclin-dependent kinase 8 (CDK8) is a promising target due to its role in regulating transcription via the TGF-β/Smad pathway, though CDK8 inhibitors have not been thoroughly studied for PF. This study aims to evaluate the potential of E966-0530-45418, a novel CDK8 inhibitor, in mitigating PF progression and explores its underlying mechanisms. We discovered that CDK8 is upregulated in lung tissues from idiopathic pulmonary fibrosis patients and in a bleomycin-induced PF mouse model. Our study further revealed that E966-0530-45418 inhibits PF progression by attenuating the activity of the transcription factor Smad3, which is involved in TGF-β1/Smad signaling, along with RNA polymerase II to downregulate fibrosis-associated protein expression in alveolar epithelia and lung fibroblasts and consequently mitigate myofibroblast differentiation and collagen deposition. E966-0530-45418 also blocks STAT3 signaling to obstruct M2 macrophage polarization, further suppressing PF progression. Moreover, E966-0530-45418 administration ameliorated lung function deterioration and lung parenchymal destruction in the bleomycin-induced PF mouse model. These findings indicate that E966-0530-45418 holds promise as a pioneering CDK8 inhibitor for treating PF.

## Introduction

Pulmonary fibrosis (PF), particularly idiopathic pulmonary fibrosis (IPF) being the most common interstitial lung disease and unknown cause, is an irreversible, progressive chronic lung disease characterized by abnormal tissue repair responses that result in thickening and stiffening of the alveolar walls which severely impedes gas exchange. This condition manifests with symptoms such as cough and dyspnea and, unfortunately, a median survival of 3-5 years after diagnosis 1, 2. Moreover, there has been renewed interest in improving treatments for pulmonary fibrosis as its prevalence has increased with the emergence of COVID-19 3. Although FDA-approved drugs such as nintedanib and pirfenidone can improve lung function in IPF patients, they do not significantly improve quality of life or halt disease progression 2. Therefore, new therapeutic options for PF are urgently needed.

The primary pathophysiological mechanism of PF involves the activation of both lung epithelial cells through epithelial-to-mesenchymal transition (EMT) and lung fibroblasts via fibroblast-to-myofibroblast transition (FMT), a process in which the profibrotic cytokine transforming growth factor β1 (TGFβ1) plays a pivotal role. These lead to myofibroblast differentiation and the excessive production of extracellular matrix (ECM) components, particularly collagen. While this process initially promotes wound healing, an excess of EMT and FMT processes contributes to aberrant tissue repair 4. During this process, the expression of fibrotic markers, including α-smooth muscle actin (α-SMA) and collagen I, is upregulated, typically mediated by TGFβ1/Smad signaling 5-7. Other studies have shown that the polarization of M2 macrophages enhances TGFβ1 secretion and exacerbates lung fibrosis progression 8, 9. However, prior research has reported that TGFBR2 knockout leads to tumor invasion and the disruption of tissue architecture in epithelial cells 10, and TGFβRI inhibitors have been associated with cardiac toxicity 11. Consequently, TGFβ1 and its receptors are not considered viable therapeutic targets due to the risk of unpredictable adverse effects, and alternative targets for treating PF are needed.

The cyclin-dependent kinase (CDK) family plays a crucial role in mediating cellular physiological functions, given their integral connection to gene transcription and cell division 12. While some CDKs, such as CDK7, 8, and 9, do not directly impact the cell cycle, they specifically regulate gene transcription. Notably, CDK8/19 augments the expression of normally silenced genes through interactions with diverse transcription factors rather than solely regulating gene transcription during normal cell growth 13. The activity of CDK8, a serine-threonine protein kinase, is controlled by cyclin C and two regulatory subunits, MED12 and MED13, which are members of a subfamily of Mediator complexes; together, these proteins form a Mediator kinase module that interacts with transcription factors and promotes the phosphorylation of the C-terminal domain of RNA polymerase II at the serine 2/5 site, thus positively regulating transcriptional activity in the cell nucleus 14-16. CDK8 facilitates cell migration in pancreatic cancer by elevating the expression of Snail, a pivotal activator of EMT 17. Furthermore, EMT induction through the TGFβ1/Smad signaling pathway involves the regulation of gene transcription by CDK8, along with the transcription factor Smad3, cofactors, and other DNA-binding partners 18. Conversely, the inhibition of CDK8 has been shown to impede STAT3 phosphorylation and M2 macrophage polarization in metastatic breast cancer 19. CDK19, a CDK8 paralog, interacts similarly with cyclin C and Mediator. Given their sequence similarity, inhibitors targeting CDK8 also affect CDK19, resulting in dual inhibition of CDK8/19 20. Encouragingly, two selective CDK8/19 inhibitors, RVU120 and BCD-115, are undergoing clinical trials for cancer therapy 21, 22, and the adverse effects of some CDK8/19 inhibitors have been revealed to be due to the off-target impacts and improper dose selection 23. Moreover, studies have demonstrated that CDK8/19 inhibitors are well tolerated in mice 24. Collectively, these findings underscore the potential of CDK8 kinases as promising targets for treating PF. However, no CDK8 inhibitors have been explored for this disease.

Thus, we sought to test the effect of a novel, highly selective CDK8 inhibitor in PF. In previous research, we developed a promising compound, E966-0530-45418, with potent CDK8 inhibitory activity (IC_50_ of 129 nM) inhibiting EMT-related protein expression and cell migration in A549 cells 25. Here, we further assessed the attenuation of fibrosis factors and elucidated the underlying mechanisms of E966-0530-45418 *in vitro*. Additionally, we aimed to evaluate the potential for E966-0530-45418 to ameliorate pulmonary fibrosis *in vivo*, striving to develop a robust therapeutic strategy for treating PF. Results presented that E966-0530-45418 attenuated myofibroblast formation and collagen deposition in lung cells through inhibition of TGFβ1/Smad signaling and reduced M2 macrophages polarization by obstacle of STAT3 signaling; moreover, E966-0530-45418 ameliorated lung function decline in bleomycin-induced PF mouse model. These findings suggest E966-0530-45418 would be a promising therapeutic strategy against PF.

## Materials and Methods

### Materials

Prof. Wei-Jan Huang provided E966-0530-45418 at a purity greater than 97%, as determined by HPLC analysis 25 (dissolved in DMSO at 100 mM). DMSO was purchased from Sigma-Aldrich (St Louis, MO, USA). Primary antibodies against N-cadherin (#13116), Snail (#3879), Smad3 (#9523), p-RNA polymerase II (S2/5) (#13546), MED12 (#14360), and histone H3 (#9715) and labeled anti-rabbit (#7074) and anti-mouse (#7076) IgG-HRP secondary antibodies were purchased from Cell Signaling Technology (Danvers, MA, USA). Primary antibodies against STAT3 (#2281-1), p-STAT3 (S727) (#1121-1), and p-STAT3 (Y705) (#2236-1) were obtained from Abcam (Cambridge, MA, USA), while those against E-cadherin (A20798), COL1A1 (A16891), p-Smad3 (T179) (AP0554), p-Smad3 (S423/425) (AP0272), RNA polymerase II (A11181), cyclin C (A6545), arginase I (A4923), TGFβ1 (A2124), and β-actin (AC026) were obtained from ABclonal (Woburn, MA, USA). CDK8 (GTX110495), α-SMA (GTX100034), and α-tubulin (GTX112141) primary antibodies and anti-rabbit IgG secondary antibodies labeled with DyLight 594 (GTX213110-05) and DyLight 488 (GTX213110-04) were purchased from GeneTex Inc. (Hsinchu, Taiwan). The primary antibody against Pin 1 (sc-46660) was obtained from Santa Cruz Biotechnology (Dallas, Texas, USA). Recombinant human TGFβ1 was obtained from PeproTech (Cranbury, NJ, USA). pcDNA3 *CDK8-HA* (P#634) was a gift from Matija Peterlin (Addgene plasmid #14649; http://n2t.net/addgene:14649; RRID: Addgene_14649); the 7TFP *CDH1* reporter was a gift from Bob Weinberg (Addgene plasmid #91704; http://n2t.net/addgene:91704; RRID: Addgene_91704); and the pGL3-*TGFB1* reporter was a gift from Yuh-Shan Jou (Addgene plasmid #101762; http://n2t.net/addgene:101762; RRID: Addgene_101762). TurboFect transfection reagent (R0531) was obtained from Thermo Fisher Scientific (Waltham, MA, USA). Senexin A (HY-15681) and Pirfenidone (HY- B0673) were purchased from MedChemExpress (Monmouth Junction, NJ, USA).

### Cell culture

The human lung epithelial cell line A549, human lung fibroblast cell line WI-38, and monocyte cell line THP-1 were purchased from the Bioresource Collection and Research Center (Hsinchu, Taiwan). Human primary alveolar epithelial cells (AECs) were purchased from Cell Biologics (Chicago, USA, H-6053). A549 cells were cultured in Dulbecco's modified Eagle's medium (DMEM), and WI-38 cells were cultured in Eagle's minimum essential medium (MEM), both of which were supplemented with 10% fetal bovine serum (v/v), sodium pyruvate (1 mM), penicillin (100 units/mL) and streptomycin (100 μg/mL). THP-1 cells were cultured in Roswell Park Memorial Institute (RPMI) 1640 medium supplemented with 10% fetal bovine serum (v/v), glucose (2.5 g/L), sodium pyruvate (1 mM), 2-mercaptoethanol (0.05 mM), penicillin (100 units/mL) and streptomycin (100 μg/mL). M0 macrophages were generated by incubating THP-1 cells with 200 ng/mL PMA for 36 h. Human primary AECs were cultured in a complete human epithelial cell medium (Cell Biologics, Chicago, USA, H6621). All cells were incubated at 37 °C in a humidified atmosphere with 5% CO_2_.

### Public dataset analysis

To search for differentially expressed genes (DEGs), clinical transcriptomic datasets were accessed via the online database repository Gene Expression Omnibus (GEO; www.ncbi.nlm.nih.gov/geo/). Two datasets (GSE49072, n = 84; GSE110147, n = 38; nonspecific interstitial pneumonia data were excluded) were analyzed through GEO2R, which is a built-in function in GEO, to acquire the DEGs (*p* < 0.001), and were also downloaded to evaluate the mRNA levels of the queried genes across different IPF patient groups. The determination of statistical significance and the establishment of dot plots were performed using GraphPad Prism 6.01 software. Another online database, PHAROS (https://pharos.nih.gov/targets), was used to find TChem targets at the target development level. The online database Reactome (https://reactome.org/) was used to identify proteins involved in signaling via the TGFβ receptor complex in humans. The intersection of the DEGs from the two GEO datasets, the TChem targets, and the proteins involved in TGFβ1 signaling was determined through a Venn diagram analysis on the VENNY 2.1 website (https://bioinfogp.cnb.csic.es/tools/venny/). A heatmap reflecting the intersecting gene expression in the GSE110147 dataset was generated via Heatmapper (http://www.heatmapper.ca/).

Spearman correlation analysis in the GSE110147 dataset was performed using GraphPad Prism 6.01 software to evaluate the coefficient (r) and *p* value. The colored grid chart with the obtained coefficient (r) and *p* value was generated by Microsoft PowerPoint.

The expression patterns of whole signaling pathways or protein complexes were assessed through Gene Set Enrichment Analysis (GSEA) software version 4.2.2. Patient samples in the GSE49072 and GSE110147 datasets obtained from the GEO database were manually sorted into two groups (normal and IPF patients) and then imported into the program for gene ranking.

Kaplan-Meier graphs were generated with the online KM plotter tool (www.kmplot.com). Information on the time until death for patients from dataset GSE28221 (n = 75) was plotted. Gene expression cutoffs to define the low- and high-expression groups were determined by Cox proportional hazards regression analysis, and the best-performing cutoff was selected for the final analysis.

The STRING online database (https://string-db.org/) was utilized to analyze protein interactions involved in TGFβ1/Smad or IL6/STAT3 signaling. In the STRING online database, the network was set to the cutoff (interaction score >0.4 for TGFβ1/Smad signaling and >0.15 for IL6/STAT3 signaling) and subdivided into differently colored nodes by k-means clustering with the default inflation parameter 3.0.

### Molecular docking and interaction analysis

The CDK8 protein structure (PDB ID: 4F6W) was obtained from the Protein Data Bank (PDB) repository. The modeling software LeadIT version 2.3.2 (BioSolveIT, GmbH, Sankt Augustin, Germany) was used to perform the molecular docking study. The CDK8-binding site has a radius of 10 Å and is centered on the cocrystal ligand. The compound used for docking was prepared by adding relevant charges and obtaining its 3D conformation. The FlexX module within LeadIT was used for docking. Molecular docking was performed using the default settings. Interactions between docking poses were analyzed using LeadIT and BIOVIA Discovery Studio.

### Cytotoxicity assay

Cytotoxicity was assessed through the MTT assay. Four cell types were tested, specifically, A549 (5×10^3^ cells), THP-1 (5×10^3^ cells), WI-38 (1×10^4^ cells), and human primary AECs (1×10^4^ cells). The indicated number of cells in 100 μL of medium were treated with vehicle or the test compound in 96-well plates for 12, 24, or 48 h. Then, 0.5 mg/mL MTT was added, and the plates were incubated for an additional 1 h at 37 °C. The cells were then pelleted and lysed in 100 μL of dimethyl sulfoxide, and the absorbance at 550 nm was measured with a microplate reader.

### Flow cytometric analysis

Human primary AECs (2×10^5^ cells/well) were seeded into 6-well plates and incubated until they reached 90% confluence. After treatment, the cells were collected with 0.25% trypsin for 3 min at 37 °C, washed with phosphate-buffered saline (PBS), and diluted to a concentration of 5×10^9^/L. For cell cycle analysis, the cells were fixed with cold 75% alcohol for 30 min at 4 °C. After centrifugation, the fixed cells were washed with cold PBS and incubated with propidium iodide (PI) (0.1% Triton X-100, 100 μg/mL RNase A, and 80 μg/mL PI in PBS) for 30 min. For protein expression, the cells were either fixed and permeabilized with cold 100% methanol for 30 min at 4 °C (for p-Smad3 T179) or permeabilized with 0.1% Triton X-100 for 15 min at room temperature (RT) and then fixed with 2% formaldehyde for 15 min (for CDK8) and then blocked with Fc blocking human IgG (1 μg of IgG for 10^6^ cells, Miltenyi Biotec, Auburn, CA, USA, 130-059-901) for 15 min at RT, followed by incubation with a primary antibody (5×10^5^ cells/100 µL; anti-p-Smad3 T179, 1:100; anti-CDK8, 1:100) for 30 min at RT, followed by incubation with a secondary antibody (Dylight488, 1:100) for 20 min at RT in the dark.

The THP-1 cells (1×10^6^ cells/well) were seeded into 6-well plates. After treatment, the cells were collected with 0.5 mM EDTA, washed with PBS, and blocked with Fc blocking human IgG for 15 min at RT, followed by incubation with a FITC-conjugated anti-human CD206 antibody (5 µl for 10^6^ cells in 100 µl; BioLegend, San Diego, California, USA; 321104) for 30 min at RT in the dark.

A FACScan flow cytometer and Cell Quest software (Becton Dickinson, Mountain View, CA, USA) were used to analyze the cell cycle distribution and the expression of the indicated proteins.

### Immunoblotting and immunoprecipitation

Cells and mouse lung tissues were homogenized using lysis buffer supplemented with protease inhibitor cocktail (Millipore) and phosphatase inhibitor and centrifuged at 4 °C for 30 min at 17,000g, after which the supernatant was mixed with 4X protein loading buffer, and the mixture was boiled at 100 °C for 5 min. The protein samples were subjected to SDS-PAGE transferred to PVDF membranes and blocked with 5% skim milk in TBST for 1 h at RT. Immunoblotting was performed overnight with primary antibodies in Tris-buffered saline + Tween 20 (TBST) at 4 °C, followed by incubation with HRP-conjugated secondary antibodies for 1 h at RT. Bound antibodies were detected using enhanced chemiluminescence (ECL) reagent and exposure to photographic film. In the immunoprecipitation assay, cell or tissue lysates were immunoprecipitated with 1 µg of mouse anti-CDK8 antibody (Santa Cruz, Dallas, Texas, USA, sc-13155, 1:20) and Protein A/G MagBeads (Genscript, Piscataway, NJ, USA, L00277) overnight at 4 °C. After the precipitated beads were washed three times with 1 mL of PBS, the bound immune complexes were separated by 10% or 12% SDS-PAGE and analyzed by immunoblotting.

Cytoplasmic and nuclear protein fractionation of the cells was performed using a kit (GoalBio, Taipei, Taiwan, W-7883) following the manufacturer's instructions prior to immunoblot analysis.

### RNA isolation and real-time qPCR

Total RNA was isolated from cells using TRIzol reagent (Invitrogen, 15596018). Single-strand cDNA used for PCR was synthesized from 2 μg of total RNA using the PrimeScript™ RT Reagent Kit (TaKaRa, San Jose, CA, USA, RR037). Real-time quantitative PCR (RT‒qPCR) was performed using a StepOne™ Real-Time PCR System (ABI) in a total reaction volume of 10 μL per reaction, consisting of 5 μL of SYBR Green PCR Master Mix (Applied Biosystems, A25741), 2.5 pmol each of forward/reverse primer, and 1 μL of cDNA. The oligonucleotide primers used for amplification are shown in the Supplementary Table S1. GAPDH served as the endogenous control to normalize variations in total RNA levels in each sample. The threshold cycle (Ct) in the exponential phase of amplification was detected, the relative mRNA expression level was determined by calculating the ΔΔCt values, and the fold change was expressed as 2^-ΔΔCt^. The value of each control sample was set at 1 and was used to calculate the fold change in the expression of target genes.

### Immunofluorescence staining

A549 and WI-38 cells (5×10^5^ cells/well) were seeded into 6-well plates with a coverslip, and human primary AECs (1×10^4^ cells/well) were seeded into opaque 96-well plates (Greiner, Kremsmünster, Germany, 655090) and cultured until they reached 90% confluence. After treatment, the cells were fixed with 4% paraformaldehyde in PBS for 15 min and subsequently washed three times with PBS (5 min/wash), followed by permeabilization with 0.05% saponin in PBS for 10 min at RT (except for the cells used for detection of the CDK8 protein, which were permeabilized using 0.1% Triton X-100 in PBS). The cells were rinsed twice with PBS for 5 min, blocked with 1% BSA and 22.52 mg/ml glycine in PBS for 30 min at RT, and then incubated with primary antibodies, including rabbit anti-COL1A1 (1:200), anti-α-SMA (1:500), anti-N-cadherin (1:400), anti-E-cadherin (1:200) or anti-CDK8 (1:200), at 4 °C overnight, followed by a 1 h incubation at RT with DyLight 488- or DyLight 594-conjugated anti-rabbit IgG secondary antibodies (1:500). Mounting medium containing DAPI stain was dropped onto the slides, and the cover slides were applied, except in the case of the human primary AECs, in which the nuclei were stained with 0.3 µg/mL DAPI in PBS for 3 min, followed by washing in PBS. Images of A549 and WI-38 cells were acquired with a Zeiss LSM880 confocal microscope, and images of human primary AECs were acquired with an ImageXpress Micro confocal microscope.

### Wound healing and Transwell migration assays

A549 cells (2×10^5^ cells/well) were seeded into 12-well plates and grown to 90% confluence; then, the cellular monolayer was wounded with a sterile 10 μL pipette tip and washed with culture medium to remove detached cells, followed by incubation with serum-free medium in the presence or absence of test compounds and control reagents for 1 h, subsequently with or without TGFβ1 (10 ng/mL) for an additional 24 h at 37 °C in a 5% CO_2_ incubator. In addition, cell migration was measured in an assay using 24-well (8 µm pore size) Transwell inserts (Corning, NY, USA, 3464). A549 cells (5×10^4^ cells/well) were seeded into the upper chamber in a serum-free medium, while the lower chamber contained 10% FBS in the complete medium as a chemoattractant. Medium with or without TGFβ1 (10 ng/mL) and with or without the test compounds and control reagents were added to each well for 18 h, and then the medium and the cells in the upper chamber were removed, after which the cells in the lower chamber were stained with 0.2% crystal violet. Images of wound healing and Transwell assay plates were taken through a Nikon microscope (Nikon, Tokyo, Japan) with a 4× objective. The percent wound closure and the number of cells in the lower chamber of the Transwell were determined using ImageJ software (National Institutes of Health, Bethesda, MD, USA).

### Chromatin immunoprecipitation (ChIP)

After treatment, the A549 cells were subjected to ChIP assays (Cell Signaling, #9003). In the ChIP procedure, chromatin was digested with 1 µL of micrococcal nuclease, followed by nuclear envelope lysis via 40% amplitude pulses for 4 sets of 15-sec pulses with a 10-sec break between pulses on ice using a Branson Sonifier 150, and immunoprecipitation with an anti-Smad3 antibody (Cell Signaling Technology, #9523, 1:50). Subsequently, RT‒qPCR was performed as described above with 1 μL of DNA fragments. The threshold cycle (Ct) in the exponential phase of amplification was detected, and the DNA fragment expression level was determined by calculating the percent input (2% x 2^(C[T] 2%Input Sample - C[T] IP Sample)^).

### Luciferase reporter assay

A549 cells (5×10^5^ cells/well) were seeded into 6-well plates for 24 h and then transfected with different luciferase reporter plasmids for 6 h using Turbofect transfection reagent (Thermo Fisher) following the manufacturer's instructions, after which they were treated with the test compounds and TGFβ1 (10 ng/ml) for 24 h. The firefly luciferase activity of the transfected cells was assayed with a luminometer and the Luciferase Assay System (Promega) according to the manufacturer's instructions. A *Renilla* luciferase expression vector (hRluc/TK: 0.1 μg) was used as a control for transfection efficiency in these experiments.

### *In vivo* experimental model

Mice were purchased from BioLASCO Taiwan Co., Ltd. Mice were housed in 3-5 per cage in a controlled environment; 12 h light/dark cycle, with specific-pathogen free (SPF) microisolators, maintaining an air temperature range of 22 ± 2℃ and a humidity of 55 ± 10% and given food and water *ad libitum*. The animal experiments were performed according to the relevant guidelines and regulations following ethical standards, and the protocols were reviewed and approved by the Institutional Animal Care and Use Committee of the National Taiwan University College of Medicine (IACUC number 20210297).

Eight-week-old male C57BL/6 mice received intratracheal (I.T.) instillation of bleomycin (Nippon Kayaku) at a dose of 5 mg/kg diluted in PBS or PBS only (sham) after anaesthesia with Zoletil™ (50 mg/kg; Virbac Co., Ltd, Carros, France) on day 0. The mice were treated with 50 mg/kg E966-0530-45418 suspended in normal saline on day 0 (before I.T. bleomycin installation) or day 7 by oral administration once daily (q.d.). Body weight was measured once daily. Lung function was evaluated in unrestrained mice using whole-body plethysmography (WBP) (BUXCO; EMKA Technologies, Paris, France) at the Taiwan Mouse Clinic on day 21. Enhanced pause (Penh) and peak expiratory rate (PEF) values were calculated as *in vivo* airway obstruction indices. *In vivo* micro-CT scanning (Skyscan 1176, Bruker, Belgium) was conducted in a laboratory animal center at the National Taiwan University College of Medicine on day 22. Image reconstruction, ring artifact, and beam-hardening correction were performed using GPU-Nrecon software. Reconstructed cross-sections were reoriented, and the region of interest (ROI) was further selected. The analysis was performed using standard micro-CT methods from Bruker. The lung volume was isolated and further quantified using CTAn software (1.20.8). On day 22, the mice were sacrificed, and bronchoalveolar lavage fluid (BALF) and lung samples were collected for further analysis.

BALF cells (5×10^6^ cells/mL) from mouse lungs were fixed and permeabilized with cold 90% methanol in PBS for 30 min at 4 °C and then blocked with Fc-blocking mouse IgG (1 μg of IgG for 10^6^ cells, Miltenyi Biotec, 130-092-575) for 15 min at RT, followed by incubation simultaneously with PE-conjugated anti-mouse CD206 antibody (141706) and FITC-conjugated anti-mouse TGFβ1 antibody (141414) (5 µg of both for 10^6^ cells in 100 µl, BioLegend) for 30 min at RT in the dark followed by conducting flow cytometric analysis.

### Immunohistochemical analysis

Left lung tissues from the mice were embedded in paraffin and sectioned (5 μm thickness). The tissue sections were dewaxed and rehydrated. Antigen retrieval was performed by autoclaving the slides in Trilogy solution (Cell Marque, Hot Springs, AR) at 121 °C for 10 min. The slides were blocked with 3% H_2_O_2_ and 5% fetal bovine serum, incubated with primary antibodies, including anti-COL1A1 (1:100), anti-α-SMA (1:200, Abcam, ab5694), anti-N-cadherin (1:50) or anti-p-Smad3 T179 (1:100), at 4 °C overnight, and then treated with polymer-HRP reagent (Dako Cytomation, Glostrup, Denmark). Peroxidase activity was visualized using a diamino-benzidine tetrahydrochloride solution (DAKO), and the sections were counterstained with hematoxylin. Dark brown nuclear staining was defined as positive; no staining was defined as negative.

### Statistical analysis

Statistical analysis and data plotting were performed using Prism 6.01 (GraphPad software). The data are expressed as the mean ± SEM. The sample size (n) for each statistical analysis is specified in the corresponding figure legends. Two-tailed unpaired t-tests were used to analyze the differences between the two groups. One-way or two-way ANOVA was used when more than two groups were compared. When ANOVA showed significant differences between groups, Tukey's post hoc test was used to determine the pairs of groups showing statistically significant differences. A *p* value < 0.05 was considered to indicate statistical significance.

## Results

### CDK8 expression and TGFβ signaling are enhanced during IPF progression

**Figure 1:**
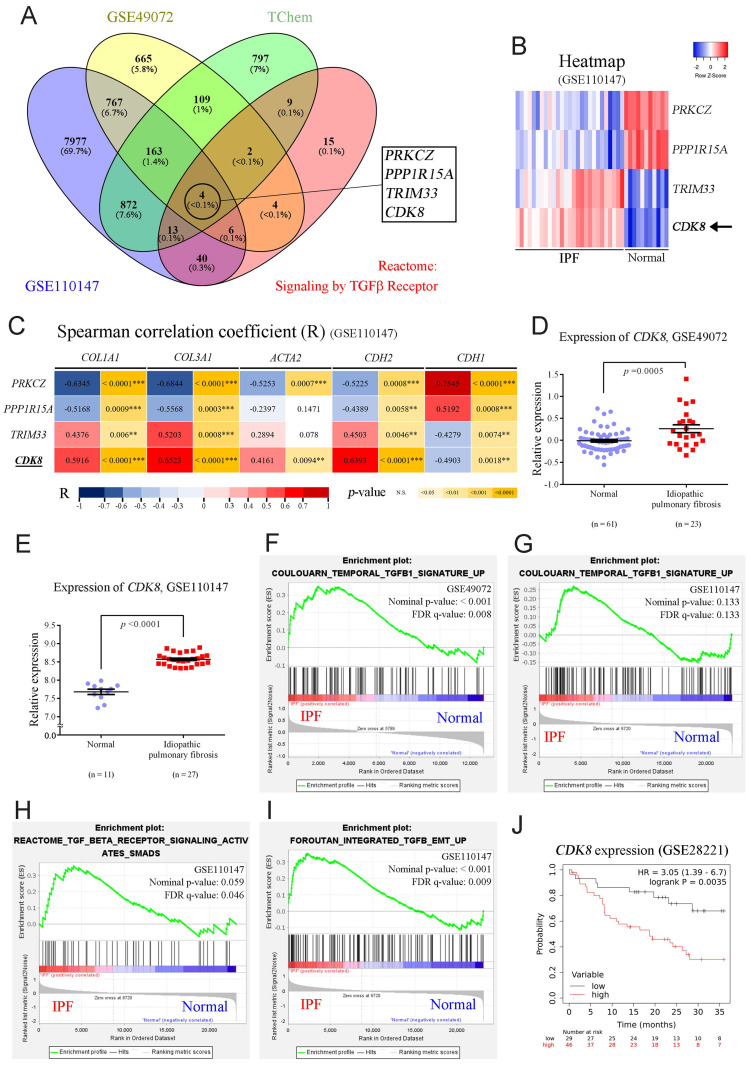
** CDK8 overexpression and active TGFβ signaling were observed in pulmonary fibrosis patients.** (**A**) Venn diagram showing the four genes in the intersection of the differentially expressed genes between human normal and IPF lung samples in two microarray datasets (GSE49072 and GSE110147), the druggable proteins in the TChem dataset, and the proteins involved in TGFβ signaling in the Reactome dataset. (**B**) Heatmap of the four intersecting genes, showing the differences in their expression between normal controls and IPF patients in the GSE110147 dataset. The color indicates the z score ranging from dark red for the most significant upregulation in IPF to dark blue for the greatest downregulation in IPF. (**C**) Correlation of the four intersecting genes expression with that of genes encoding fibrosis-related proteins (*COL1A1*, *COL3A1*, *ACTA2*) and EMT-related proteins (*CDH2*, *CDH1*) in the GSE110147 dataset. The color indicates the correlation coefficient (R) ranging from dark blue for -1 to dark red for +1, and the *p*-value with yellow marked. (**D**, **E**) Comparison of *CDK8* mRNA expression in human IPF lung tissue samples and normal tissue samples from the GSE49072 (D) and GSE110147 (E) datasets. The *P* value was determined using a two-tailed Student's unpaired t-test, and the results are shown as the mean ± SEM. (**F**, **G**) GSEA of TGFβ1 signaling pathway components in human IPF lung tissue samples compared with normal tissue samples from the GSE49072 (F) and GSE110147 (G) datasets. (**H**, **I**) GSEA of TGFβ/Smad signaling pathway genes (H) and TGFβ/EMT pathway genes (I) in human IPF lung samples vs. normal lung samples from the GSE110147 dataset. The *P* value was computed using the 2-sided permutation test with the Benjamini-Hochberg adjustment for multiple comparisons. FDR: false discovery rate (F-I). (**J**) Kaplan-Meier survival plots for human IPF patients with high vs. low *CDK8* expression, based on data from the GSE28221 dataset. HR and *p* values were derived from Cox regression analysis.

To validate whether CDK8 plays a vital role in the progression of PF, we examined the overlap among four relevant datasets to identify proteins implicated in TGFβ signaling and IPF progression that are also suitable drug development targets. First, we analyzed two publicly available GEO databases of lung samples (GSE49072 and GSE110147) to identify genes with significant differential expression between IPF patients and the control group (*p* < 0.001). In parallel, we obtained a list of proteins associated with the TGFβ1 signaling pathway within the Reactome dataset, and we employed TChem in Pharos to identify potentially druggable proteins. Then, we determined the overlapping genes identified in these four analyses, which yielded four candidates: *PRKCZ*, *PPP1R15A*, *TRIM33*, and *CDK8* (**Figure 1A**). In PF, fibrotic proteins such as COL1A1, COL3A1, and α-SMA were upregulated, along with the EMT marker N-cadherin, while E-cadherin was downregulated 4, 17, 26. Therefore, *COL1A1*, *COL3A1*, *ACTA2* (encoding α-SMA), *CDH2* (encoding N-cadherin), and *CDH1* (encoding E-cadherin) were used for Spearman correlation coefficient analysis with four candidates. *PRKCZ* and *PPP1R15A* showed negative correlations, while *TRIM33* and *CDK8* exhibited positive correlations with the fibrotic markers in the GSE110147 dataset. Notably, *CDK8* demonstrated a stronger positive correlation, particularly in IPF patients, making it the most suitable target for inhibitor development in PF (**Figure 1B, C**). Additionally, analysis of RNA expression levels from alveolar macrophages in the GSE49072 dataset and lung tissue in the GSE110147 dataset revealed that *CDK8* expression was significantly greater in the IPF patient group than in the control group for both datasets (**Figure 1 D, E**).

Given the association of CDK8 with the TGFβ signaling pathway, GSEA was carried out to profile the expression of the multiprotein complex as a whole. Analysis of the GSE110147 and GSE49072 datasets revealed the enrichment of genes involved in the TGFβ1 signaling pathway in the IPF group compared to the normal group (**Figure 1F, G**). Further enrichment analysis of the GSE110147 dataset revealed the activation of TGFβ/Smad signaling and promotion of TGFβ-mediated EMT in IPF patient samples (**Figure 1H, I**). Furthermore, analysis of expression array data from 75 IPF patient samples (GSE28221) revealed that high *CDK8* RNA levels were associated with significantly lower survival times over 36 months (**Figure 1J**). These results suggest that CDK8 may be a promising target for PF treatment.

### E966-0530-45418 is not cytotoxic to lung or immune cells

**Figure 2:**
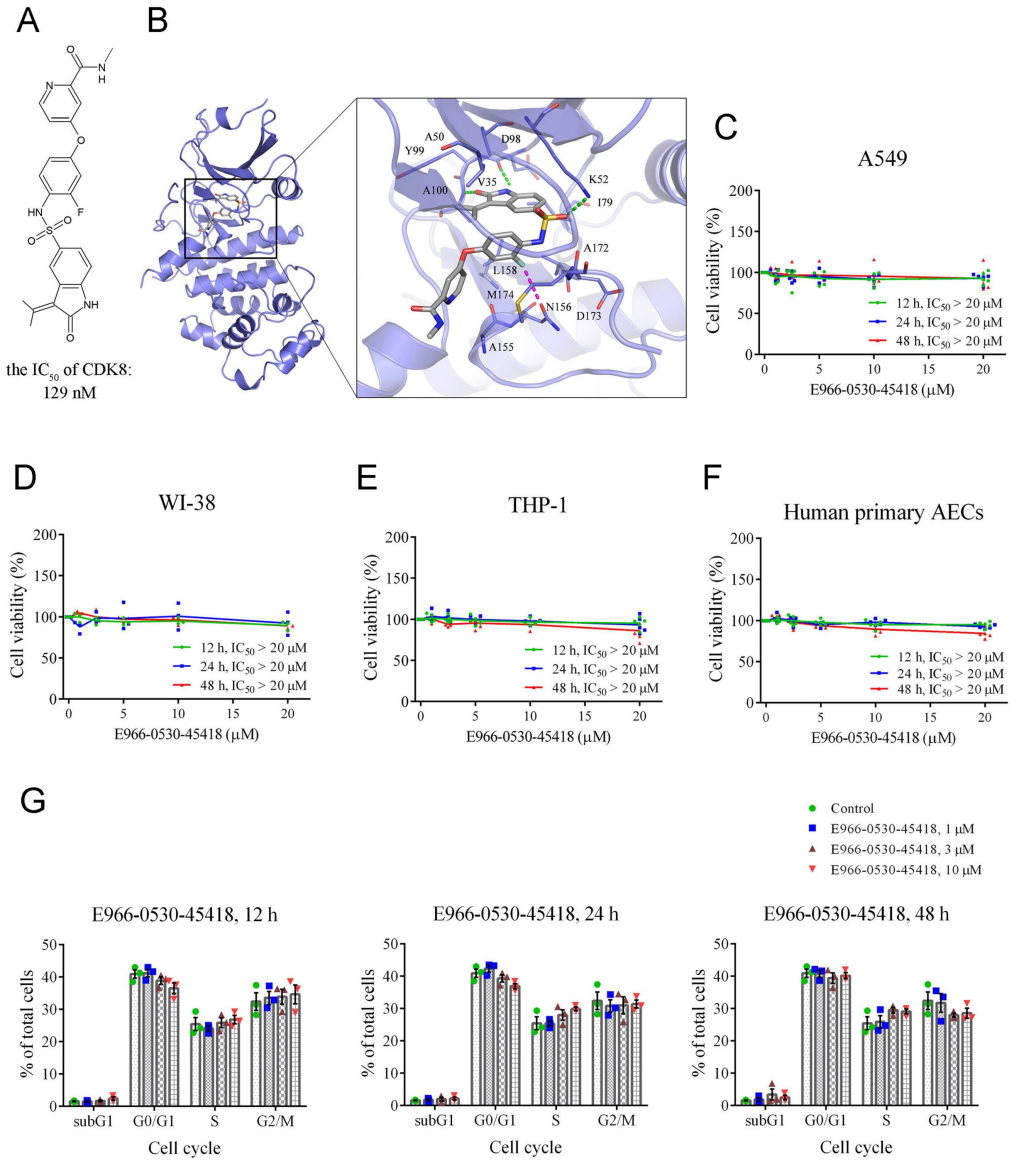
** E966-0530-45418 is not cytotoxic and does not affect cell cycle progression.** (**A**) The structure of E966-0530-45418. (**B**) Molecular docking analysis showed that E966-0530-45418 (gray) shows favorable interactions within the binding site in CDK8 (blue). The docking pose is represented as sticks. Binding site residues are rendered as lines and labeled. Halogen interactions and hydrogen bonds are denoted as purple or green lines, respectively. (**C**-**F**) Cell viability was measured by MTT assay for A549 (C), WI-38 (D), and THP-1 (E) cells and human primary alveolar epithelial cells (AECs) (F), which were incubated with different concentrations (0, 1, 2.5, 5, 10, or 20 μM) of E966-0530-45418 for 12, 24 or 48 h (n = 5 independent samples per group). The IC_50_ values were calculated by a sigmoidal dose-response equation. The results are presented as the mean, along with the individual replicates. (**G**) Flow cytometric analysis of PI staining was used to evaluate the cell cycle distribution of human primary AECs treated with or without different concentrations (1, 3, or 10 μM) of E966-0530-45418 for 12, 24, or 48 h (n = 3 independent samples per group). The results are shown as the mean ± SEM. No significant statistics were determined using two-way ANOVA.

Having shown the importance of CDK8 for PF, we next turned to CDK8 inhibitor E966-0530-45418 research to verify the compound's efficacy against PF. Our prior investigation demonstrated that E966-0530-45418 (depicted in **Figure 2A**) has potent CDK8 inhibitory activity, with an IC_50_ of 129 nM 25. To further investigate its interactions with CDK8, E966-0530-45418 was subjected to molecular docking analysis within the binding site of CDK8. E966-0530-45418 features an oxindole scaffold that establishes crucial hydrogen bonds with hinge residues D98 and A100 in CDK8 (**Figure 2B**). The preservation of these hydrogen bonds is pivotal for kinase inhibition, as highlighted in a previous screen that identified a CDK8 inhibitor with an oxindole scaffold 27. Optimizing the inhibitor did not disrupt interactions with hinge residues 25. Hydrophobic interactions in the hinge region involve residues V35, A50, I79, Y99, and L158, all contributing to hydrophobic contact with the oxindole scaffold. E966-0530-45418 also forms an additional hydrogen bond with K52 in CDK8, and a fluorine atom on the compound engages in a halogen interaction with residue N156. Further hydrophobic interactions involve residues A155 and M174 with the two terminal rings. This docking analysis revealed structural features indicative of the potential of E966-0530-45418 as a CDK8 inhibitor.

Additionally, we used an MTT assay to assess the cytotoxicity of E966-0530-45418 in four cell types: A549 human lung epithelial cells, WI-38 human fetal lung fibroblasts, THP-1 human leukemia monocytes, and human primary AECs. The results indicated that varying concentrations and treatment durations of E966-0530-45418 did not significantly affect the viability of any of these cells (**Figure 2C-F**).

Although CDK8/19 are thought to be dispensable for general transcription and normal cell cycle progression 13, given the roles of other CDKs in regulating the cell cycle 12, we sought to confirm that E966-0530-45418 would not alter the cell cycle distribution. To this end, we conducted propidium iodide (PI) staining and flow cytometric analysis of human primary AECs. The results revealed that E966-0530-45418 treatment did not significantly alter the cell cycle beyond minor variations in various phases (**Figure 2G & Supplementary Figure S1**). These results show that E966-0530-45418 possesses interactions with CDK8 and not the cytotoxicity and the influence on the cell cycle.

### E966-0530-45418 significantly reduced the expression of EMT and FMT proteins and inhibited cell migration

**Figure 3:**
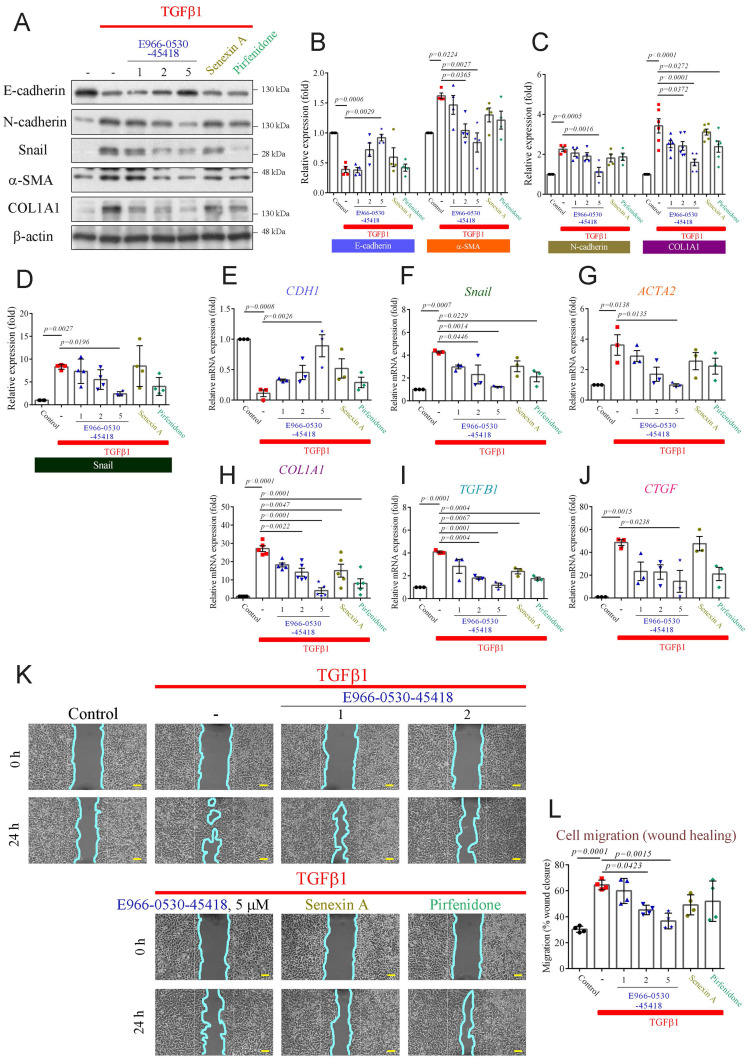
** E966-0530-45418 significantly attenuates the TGFβ1-induced increase of EMT proteins, fibrotic markers, and cell migration.** (**A**-**J**) A549 cells were exposed to the indicated concentrations of E966-0530-45418 (μM), senexin A (5 μM), pirfenidone (1 mM), or no inhibitor in the presence of TGFβ1 (10 ng/mL) for 24 h. The protein levels of E-cadherin, N-cadherin, snail, α-SMA, and COL1A1 were determined by western blot in A549 cells (n = 4 independent samples per group, except for COL1A1, where n = 6) (A-D). The mRNA levels of E-cadherin, snail, α-SMA, COL1A1, TGFβ1, and CTGF were analyzed by RT‒qPCR in A549 cells (n = 3 independent samples per group, except for COL1A1, where n = 5) (E‒J). (**K**,** L**) A549 cells were treated with E966-0530-45418 at the indicated concentrations (μM), senexin A (5 μM), pirfenidone (1 mM), or no inhibitor in the presence of TGFβ1 (10 ng/mL), allowed to migrate into the wound area for 24 h and photographed; the cyan solid line depicts the edge between the cell-occupying region and the wound area (40× magnification) (Scale bar: 100 μm) (K). Cell migration into the wound was quantified using ImageJ software (L). (n = 4 independent samples per group). The results are shown as the mean ± SEM. *P* values were determined using one-way ANOVA followed by Tukey's post hoc test (B-J, and L).

Aberrant wound healing from repeated injuries causes abnormal myofibroblast formation, progressive fibrosis, and excessive ECM deposition, culminating in lung parenchymal destruction 28, 29. Our previous study revealed that CDK8 inhibition attenuated the TGFβ-induced EMT process in cancer metastasis 30. Moreover, TGFβ1 induces EMT and FMT, promotes the secretion of TGFβ1 and connective tissue growth factor (CTGF) in lung cells, and plays a vital role in fibrosis 26, 28, 29, 31. Hence, we aimed to assess the impact of E966-0530-45418 on TGFβ1-induced myofibroblast differentiation and ECM production via EMT in human lung epithelial A549 cells. Western blot and real-time qPCR results demonstrated that TGFβ1 treatment significantly increased the protein (**Figure 3A-D**) and mRNA expression (**Figure 3E-H & Supplementary Figure S12**) of EMT markers, including N-cadherin, Snail, α-SMA, and COL1A1, and decreased the expression of E-cadherin in A549 cells (**Figure 3B, E**), indicating that TGFβ1 could induce EMT progression in our model. Treatment with 1 µM, 2 µM or 5 µM E966-0530-45418 reduced EMT marker levels in TGF-β-treated cells in a concentration-dependent manner (**Figure 3A-H**) and also limited the TGFβ1-induced increases in the mRNA levels of TGFβ1 and CTGF (**Figure 3I, J**). Further, 5 μM E966-0530-45418 treatment inhibited EMT protein and mRNA expression more potently than the commercial CDK8 inhibitor senexin A and the clinical IPF drug pirfenidone (**Figure 3A-J**). Moreover, immunofluorescence staining analysis confirmed that E966-0530-45418 blocked the TGFβ1-induced changes in the expression of E-cadherin, N-cadherin, α-SMA, and COL1A1 in A549 cells (**Supplementary Figure S2A**).

We next evaluated whether E966-0530-45418 could inhibit FMT with similar experiments in human lung fibroblast WI-38 cells. The compound also decreased the TGFβ1-induced expression of COL1A1 and α-SMA, as shown by western blot and qPCR analysis (**Supplementary Figure S3A-D**). The downregulatory effects of senexin A and pirfenidone were less pronounced than those of E966-0530-45418. Additionally, E966-0530-45418 diminished the TGFβ1-induced increases in the mRNA expression of TGFβ1 and CTGF (**Supplementary Figure S3E, F**). In addition, immunofluorescence staining results revealed that E966-0530-45418 decreased the TGFβ1-induced expression of α-SMA and COL1A1 in human lung fibroblast WI-38 cells (**Supplementary Figure S3G**).

Functionally, we next assessed whether E966-0530-45418 inhibited the migration of A549 cells through wound healing and Transwell migration assays. The results demonstrated a significant increase in wound closure at 24 h in response to TGFβ1 treatment, and E966-0530-45418 significantly impeded this TGFβ1-induced effect on migration (**Figure 3K, L**). Furthermore, we confirmed the inhibitory effect of the compounds on migration by Transwell assays, and the results showed that E966-0530-45418 treatment significantly mitigated TGFβ1-induced cell migration (**Supplementary Figure S2B, C**). These findings show that E966-0530-45418 can attenuate TGFβ1-induced myofibroblast differentiation and ECM production, both caused by epithelial cells through the EMT process and by fibroblasts via the FMT process. Our functional assay results demonstrated that E966-0530-45418 can inhibit cell migration.

### E966-0530-45418 suppressed TGFβ1/Smad signaling

**Figure 4:**
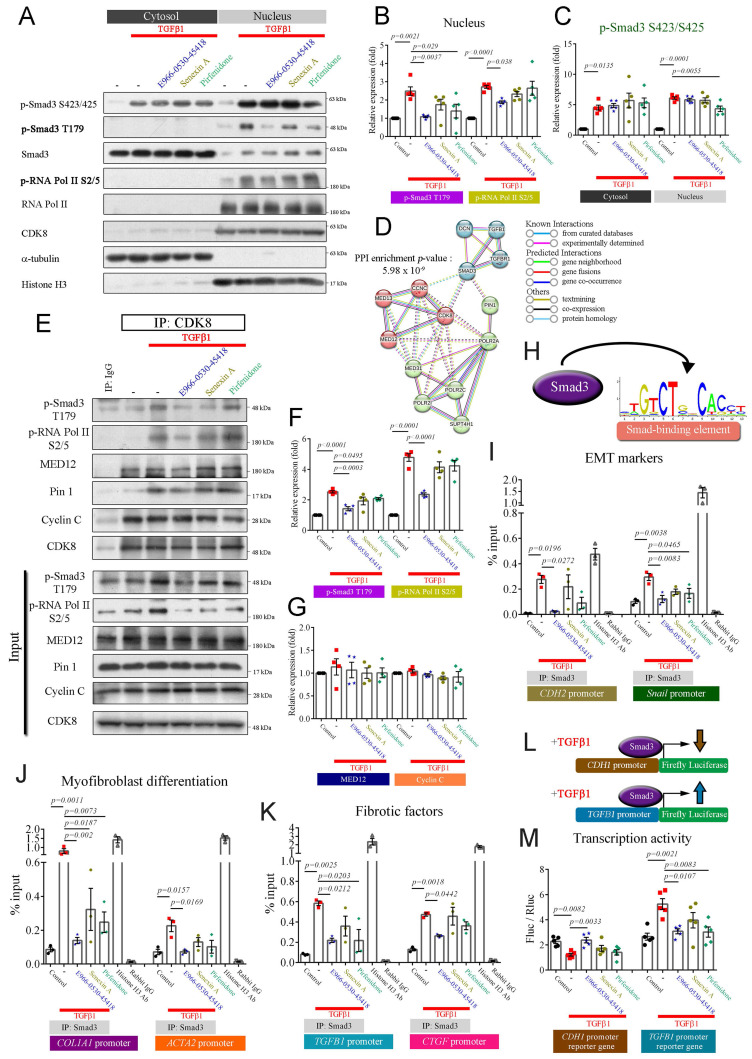
** E966-0530-45418 markedly mitigated TGFβ1/Smad3/RNA polymerase II signal transduction, subsequently lowering the transcription of EMT and fibrosis-related genes.** (**A**-**C**,** E**-**G**) A549 cells were treated with E966-0530-45418 (5 μM), senexin A (5 μM), pirfenidone (1 mM), or no inhibitor in the presence of TGFβ1 (10 ng/mL) for 3 h and then subjected to nuclear-cytosolic fractionation. The protein levels in the cytosol and nucleus were detected by western blotting and quantified (n = 5 independent samples per group) (A-C). The nuclear proteins were immunoprecipitated with an anti-CDK8 antibody and then subjected to immunoblotting to assess the interactions between CDK8 and p-Smad3 T179, p-RNA Pol II S2/5, MED12, Pin 1, and cyclin C (n = 4 independent samples per group) (E-G). (**D**) Protein-protein interaction (PPI) network analysis was established by the STRING online database. The network nodes represent proteins, and the colored edges denote evidence of interactions between different proteins in the PPI network. Strong connections and networks were clustered using the k-means cluster algorithm with default parameters and are indicated by solid lines. POLR2A: RNA polymerase II. (**H**) Schematic illustrating Smad3 binding to the Smad-binding element (sequence logo obtained from the UCSC JASPAR joint website). (**I**-**K**) A549 cells were incubated with E966-0530-45418 (5 μM), senexin A (5 μM), pirfenidone (1 mM), or no inhibitor in the presence of TGFβ1 (10 ng/mL) for 6 h and then subjected to a Smad3 ChIP assay with RT‒qPCR analysis of the promoter sequences of N-cadherin, Snail (I), COL1A1, α-SMA (J), TGFβ1 and CTGF (K). The histone H3 antibody (Ab) group was used as a positive control, and the rabbit IgG group was used as a negative control (n = 3 independent samples per group). (**L**) Schematic illustration of the human E-cadherin and TGFβ1 promoter luciferase reporter genes, the function of which might be affected by TGFβ1 treatment. (**M**) A549 cells were transfected with 7TFP *CDH1* or pGL3-*TGFB1* reporter plasmid (1 μg) for 6 h, followed by subsequent treatment with TGF-β1 (10 ng/mL) with E966-0530-45418 (5 µM), senexin A (5 µM), pirfenidone (1 mM), or no inhibitor for 24 h, and luciferase expression was subsequently determined (n = 5 independent samples per group). The results are shown as the mean ± SEM. *P* values were determined using one-way ANOVA followed by Tukey's post hoc test (B, C, F, G, I-K, and M).

Upon TGFβ1 activation, Smad2 and Smad3 undergo phosphorylation at the C-terminal domain (e.g., S423/425 on Smad3 and S465/467 on Smad2). Phosphorylated Smads and Smad4 form a complex that translocates into the nucleus 6, 32. Within the nucleus, the CDK8/Mediator complex phosphorylates Smad3 at T179, promoting high-affinity binding to the coactivator Pin1 and other transcriptional partners. This cascade leads to RNA polymerase II phosphorylation at S2/5, resulting in peak transcriptional activity and enhanced EMT and myofibroblast development 6, 15, 16, 32, 33. To elucidate whether the inhibitory effects of the compound on myofibroblast formation and migration are mediated through the repression of CDK8-associated transcriptional regulation, we investigated its impact on the canonical TGFβ1/Smad pathway. Cytoplasmic and nuclear fractions were isolated and validated using α-tubulin and histone H3 as cytoplasmic and nuclear markers, respectively. TGFβ1 treatment significantly increased the levels of phospho-Smad3 S423/425 in the cytosol and the phosphorylation of Smad3 S423/425, Smad3 T179, and RNA polymerase II S2/5 in the nuclear fraction in both cells (**Figure 4A-C & Supplementary Figure S4A-C**). E966-0530-45418 significantly reduced the levels of p-Smad3 T179 and p-RNA polymerase II S2/5 in the nucleus but did not reduce the phosphorylation of p-Smad3 S423/425 in the cytosol or nucleus in either cell (**Figure 4A-C & Supplementary Figure S4A-C**). Similar to E966-0530-45418, senexin A and pirfenidone inhibited only phospho-Smad T179 and phospho-RNA polymerase II S2/S5, specifically within the cell nucleus; however, the potencies of both agents were inferior to that of E966-0530-45418 (**Figure 4B & Supplementary Figure S4B**). These results suggested that the CDK8/Mediator complex plays a crucial role in regulating the transcription of TGFβ1/Smad signaling target genes through interactions with transcription factors and RNA polymerase II.

Next, to confirm whether E966-0530-45418 blocked the direct interaction between CDK8, Smad3, and RNA polymerase II, we used the STRING database to construct a protein-protein interaction (PPI) network to explore potential PPIs involving CDK8 that regulate TGFβ1 target gene transcription. A cluster comprising CDK8, cyclin C, MED12, and MED13 was identified (**Figure 4D**), suggesting the potential collaboration of these proteins in TGFβ1/Smad signal-mediated gene transcriptional regulation. Co-immunoprecipitation assays confirmed the interactions between CDK8, Smad3, MED12, Pin1 coactivator, and RNA polymerase II. Moreover, E966-0530-45418 treatment suppressed the TGFβ1-induced increase in the binding of CDK8 to p-Smad3 T179, Pin1, and p-RNA polymerase II S2/5 (**Figure 4E-G**). An illustration of the model of interaction based on these results is shown in **Supplementary Figure S4D**.

### E966-0530-45418 decreased the transcriptional activity of the active TGFβ1/Smad signaling pathway

Previous work has shown that Smad3 binds to Smad binding elements (illustrated in **Figure 4H**) within the promoters of target genes and that the levels of Smad3 at these promoters are increased when Smad3 is phosphorylated at T179 by CDK8, which in turn elevates the transcriptional activity of target genes 33, 34. Therefore, we used ChIP and qPCR to investigate whether E966-0530-45418 reduced TGFβ1-induced Smad3 binding to the promoters of target genes in A549 cells. TGFβ1 increased Smad3 binding to the promoters of N-cadherin, Snail, COL1A1, α-SMA, TGFβ1, and CTGF, and this effect was diminished by treatment with E966-0530-45418 (**Figure 4I-K**). In addition, we studied the effect of TGFβ1/Smad signaling on the transcriptional activity of E-cadherin and TGFβ1 via a luciferase reporter assay in A549 cells that expressing the E-cadherin or TGFβ1 promoter-reporter gene and hRluc/TK (thymidine kinase promoter-Renilla luciferase reporter plasmid, used as an internal reference). TGFβ1 treatment downregulated E-cadherin promoter activity and upregulated TGFβ1 promoter activity, and these effects were inhibited by treatment with E966-0530-45418. In addition, the ability of E966-0530-45418 to inhibit TGFβ1 promoter activity was comparable to that of pirfenidone (**Figure 4L, M**).

Next, to further verify the participation of CDK8 in TGFβ1/Smad signaling and the inhibitory effect of E966-0530-45418, we investigated the effects of CDK8 overexpression on TGFβ1/Smad signaling in cells that were or were not treated with the compound. The inhibitory effects of E966-0530-45418 were counteracted by CDK8 overexpression, which restored Smad3 and RNA polymerase II phosphorylation (**Supplementary Figure S5A-C**), indicating active TGFβ1/Smad signaling and also restored the TGFβ1-induced expression of EMT and ECM proteins (**Supplementary Figure S5D-G**). Interestingly, mere CDK8 overexpression increased the levels of p-Smad3 T179 and p-RNA polymerase II S2/5 (**Supplementary Figure S5A-C**) but did not affect the expression of EMT markers or COL1A1 (**Supplementary Figure S5D-G**). Overall, these observations indicate that TGFβ1/Smad signaling promotes the transcriptional activity of proteins associated with EMT, the ECM, and fibrosis via CDK8, leading to the increased phosphorylation of Smad3 and RNA polymerase II and that these effects are inhibited by E966-0530-45418 treatment.

### E966-0530-45418 inhibits the polarization of M2 macrophages

**Figure 5:**
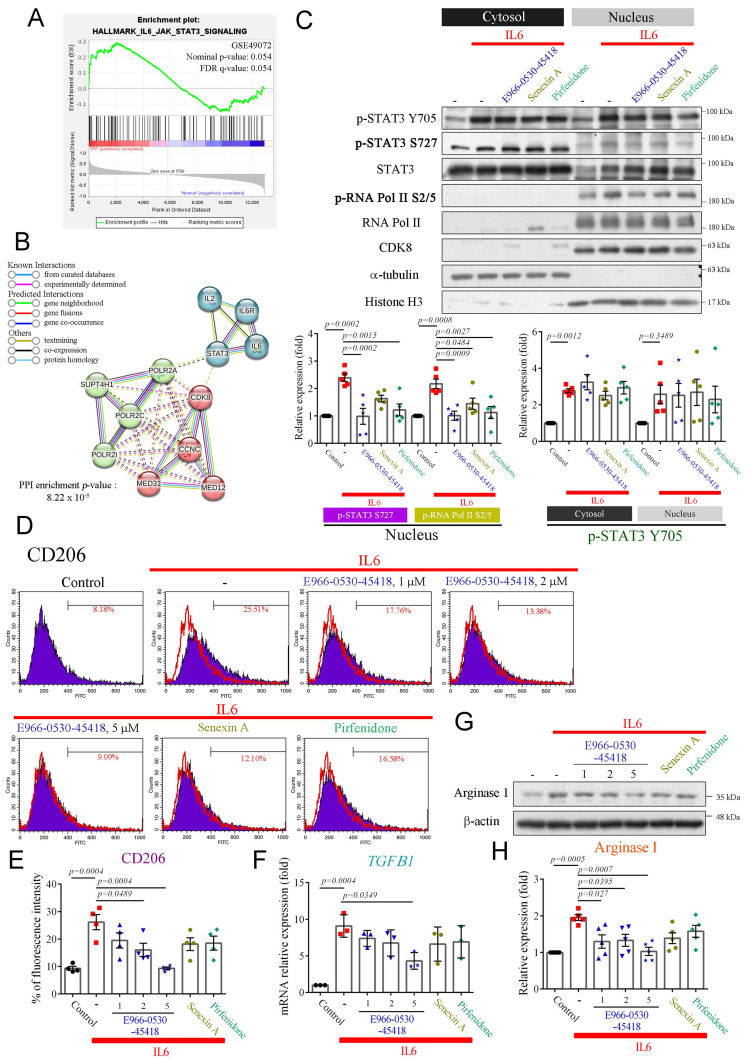
** E966-0530-45418 diminished M2 macrophage polarization induced by IL6 in PMA-treated THP-1 cells.** (**A**) GSEA of IL6/STAT3 signaling pathway genes in human IPF vs. normal lung samples from the GSE49072 dataset. The *P* value was computed using the 2-sided permutation test with the Benjamini-Hochberg adjustment for multiple comparisons. FDR: false discovery rate. (**B**) PPI network validating the interaction of IL6/STAT3/CDK8/RNA polymerase II (POLR2A) signals. Network nodes represent proteins; the colored edges denote evidence of different PPI types. Solid interactions and networks were clustered using the k-means cluster algorithm with default parameters and are indicated by solid lines. (**C**) PMA-induced THP-1 cells were treated with E966-0530-45418 (5 μM), senexin A (5 μM), pirfenidone (1 mM), or no inhibitor in the presence of IL6 (5 ng/mL) for 3 h and then subjected to nuclear-cytosolic fractionation. The protein levels in the cytosol and nucleus were detected by western blotting analysis and quantified (n = 5 independent samples per group). (**D**-**H**) PMA-induced THP-1 cells were treated with the indicated concentration of E966-0530-45418 (μM), senexin A (5 μM), pirfenidone (1 mM), or no inhibitor in the presence of IL6 (5 ng/mL) for 48 h (D and E) or 24 h (F-H). The CD206 levels were evaluated by flow cytometric analysis (n = 4 independent samples per group) (D and E). The mRNA levels of TGFβ1 were determined by RT‒qPCR (n = 3 independent samples per group) (F). The protein levels of arginase I were assessed by western blotting (n = 5 independent samples per group) (G and H). The results are shown as the mean ± SEM. *P* values were determined using one-way ANOVA followed by Tukey's post hoc test (C, E, F, and H).

Previous research has emphasized the pivotal role of M2 macrophage polarization in fibrosis, particularly through STAT3 phosphorylation at Y705 and S727 8. Phosphorylated STAT3 acts as a crucial transcription factor, activating M2 macrophages and contributing to fibrosis 35. Upon activation of the interleukin 6 (IL6)/STAT3 signaling pathway, STAT3 is phosphorylated at Y705 and S727, facilitating its dimerization, nuclear translocation, and DNA binding. Phosphorylation at S727, in particular, leads to maximal STAT3 transcriptional activity 36. Therefore, IL6 has also been identified as a critical driver of lung fibrosis 37. Furthermore, CDK8/19 inhibition reduces pSTAT3-S727 expression and attenuates M2 macrophage formation 19. Analysis of the GSE49072 dataset revealed the enrichment of genes related to the IL6/STAT3 pathway in alveolar macrophages in the IPF group compared to the control group (**Figure 5A**). Additionally, a PPI network constructed using the STRING database highlighted the clustering of CDK8 with cyclin C, MED12, RNA polymerase II, and STAT3, which may regulate gene transcription (**Figure 5B**).

We, therefore, established a macrophage polarization model to explore whether E966-0530-45418 could inhibit M2 macrophage polarization by suppressing STAT3 phosphorylation. THP-1 monocytes were differentiated into macrophages using phorbol 12-myristate 13-acetate (PMA), as confirmed by decreased CD14 expression and increased CD68 expression (**Supplementary Figure S6**); once differentiated (M0 macrophages), they were treated with IL6 to induce M2 polarization. While E966-0530-45418 did not impact the IL6-induced phosphorylation of STAT3 at Y705, it effectively suppressed its phosphorylation at S727 in the nucleus and also led to a reduction in p-RNA polymerase II S2/5 levels (**Figure 5C**). Additionally, macrophages treated with IL6 showed elevated M2 macrophage markers CD206 (**Figure 5D, E**) and mRNA levels of TGFβ1 (**Figure 5F**) and increased expression of arginase I (**Figure 5G, H**); these effects were notably diminished upon E966-0530-45418 treatment. Overall, these findings indicate that E966-0530-45418 attenuated the phosphorylation of STAT3 at S727 within the active IL6/STAT3 pathway, thereby reducing M2 macrophage polarization.

Furthermore, to investigate whether M2 macrophages contribute to lung fibrosis, the conditioned medium from IL6-treated, PMA-induced THP-1 cells was used to incubate A549 cells, as shown in **Supplementary Figure S7A**. The results displayed that the upregulation of COL1A1 and α-SMA, along with the downregulation of E-cadherin in A549 cells in response to the conditioned medium, were significantly reversed by E966-0530-45418 treatment (**Supplementary Figure S7B-F**).

### E966-0530-45418 obstructed TGFβ1-induced EMT development and ECM deposition in human primary AECs

**Figure 6:**
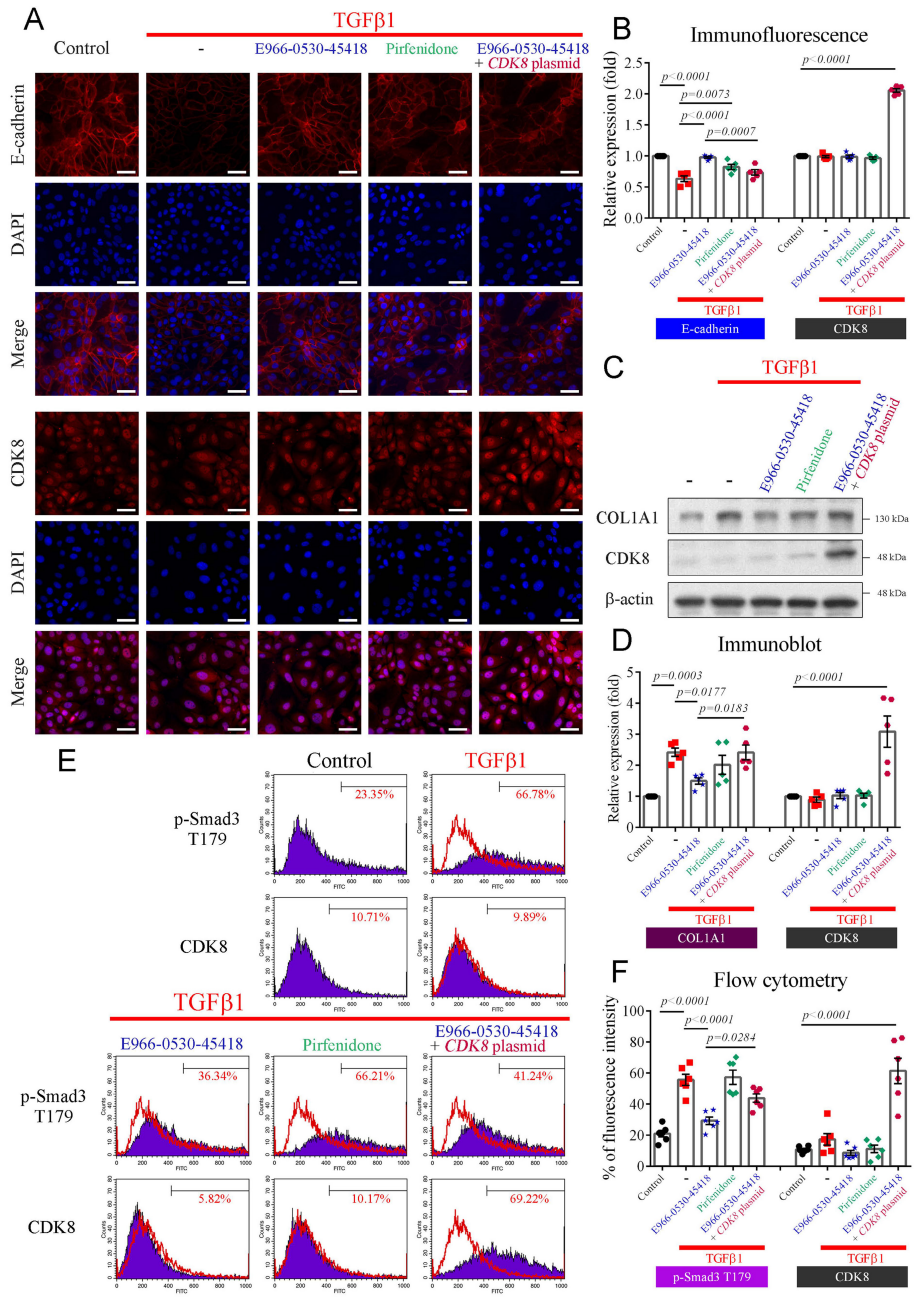
** E966-0530-45418 inhibited the activation of EMT and the collagen I protein in TGFβ1-evoked human primary AECs.** (**A**-**F**) Human primary AECs were transfected with the pcDNA3 *CDK8-HA* plasmid (1 μg) for 24 h, treated with E966-0530-45418 (5 μM), pirfenidone (1 mM) or not for 1 h, and then incubated with TGFβ1 (10 ng/mL) for an additional 24 h (A-D) or 3 h (E and F). Immunofluorescence analysis using a high-content imaging system (ImageXpress Micro confocal microscope) was used to evaluate E-cadherin and CDK8 protein expression. Images were taken at 200 × magnification (Scale bar: 50 μm) (n = 5 independent samples per group) (A and B). The protein levels of COL1A1 and CDK8 were determined by western blotting (n = 5 independent samples per group) (C and D). The protein levels of p-Smad3 T179 and CDK8 were assessed by flow cytometry (n = 6 independent samples per group) (E and F). The results are shown as the mean ± SEM. *P* values were determined using one-way ANOVA followed by Tukey's post hoc test (B, D, and F).

We further investigated whether E966-0530-45418 could impede TGFβ1-induced EMT and ECM deposition in human primary AECs. Immunofluorescence analysis revealed the conspicuous expression of E-cadherin, a pivotal protein in cell‒cell adhesion, on the cytoplasmic membrane of primary AECs. TGFβ1 stimulation promoted the EMT process, markedly decreasing E-cadherin expression (**Figure 6A, B**). However, the E966-0530-45418 treatment demonstrated a pronounced rescue effect, surpassing pirfenidone in efficacy. Notably, CDK8 overexpression counteracted the effect of the compound, decreasing E-cadherin expression (**Figure 6A, B**). Additionally, western blot analysis indicated that TGFβ1-induced the production of COL1A1, a key fibrotic protein, in primary AECs (**Figure 6C, D**). E966-0530-45418 treatment significantly attenuated COL1A1 expression, again outperforming pirfenidone, and CDK8 overexpression reversed the inhibitory impact of E966-0530-45418 (**Figure 6C, D**).

Further investigation revealed that CDK8-mediated phosphorylation of Smad3 at T179 enhances transcriptional activity in the TGFβ1/Smad signaling pathway. Flow cytometric analysis demonstrated that TGFβ1 treatment increased the level of p-Smad3 T179 in primary AECs (**Figure 6E, F**). E966-0530-45418 effectively blocked the TGFβ1-induced phosphorylation of Smad3 at T179, while pirfenidone had no such counteractive effect. CDK8 overexpression impeded the efficacy of E966-0530-45418, promoting the expression of p-Smad3 T179 (**Figure 6E, F**). Our findings collectively verified that E966-0530-45418 suppresses EMT, diminishes COL1A1 production, and mitigates ECM deposition in human primary AECs. These effects parallel those observed in A549 cells and are attributable to the inhibition of Smad3 phosphorylation at T179 by E966-0530-45418.

### E966-0530-45418 ameliorated bleomycin-induced pulmonary fibrosis* in vivo*

**Figure 7:**
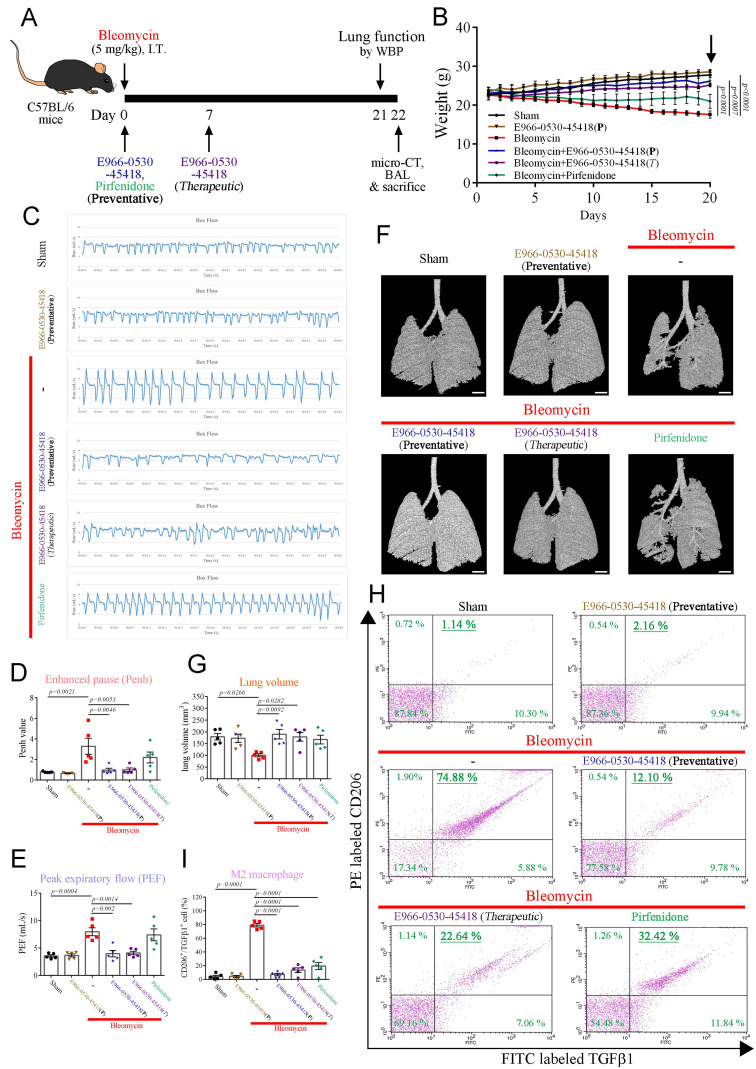
** E966-0530-45418 improved lung function in mice with bleomycin-induced pulmonary fibrosis.** (**A**) Illustration of the experimental design. 8-week-old male C57BL/6 mice were or were not administered 50 mg/kg E966-0530-45418 by oral gavage on day 0 (preventative) or day 7 (therapeutic) after bleomycin treatment through intratracheal instillation. Lung function tests and micro-CT imaging were conducted on days 21 and 22 after bleomycin treatment, respectively. Subsequently, the mice were sacrificed, and BALF and lung tissue were collected on day 22 (n = 5 independent animals per group). (**B**) Changes in body weight were recorded after bleomycin treatment (n = 5 independent animals per group). (**C**-**E**) Barometric plethysmography was conducted to evaluate pulmonary respiratory function in different groups of mice on day 21. Enhanced pause (Penh) and peak expiratory rate (PEF) values were calculated as *in vivo* airway obstruction indices (n = 5 independent animals per group). (**F**, **G**) Representative micro-CT images of lung tissues collected from mice on day 22 after bleomycin treatment. The lung architecture was assessed by micro-CT imaging and lung volume quantification (n = 5 independent animals per group) (Scale bar: 1 mm). (**H**, **I**) Flow cytometric analysis of the BALF collected on day 22 with double staining for CD206 and TGFβ1 was performed to evaluate M2 macrophage polarization (n = 5 independent animals per group). The results are shown as the mean ± SEM. *P* values were determined using one-way ANOVA followed by Tukey's post hoc test (B, D, E, G, and I).

To evaluate the potential clinical application of E966-0530-45418 in PF patients, we then assessed the *in vivo* efficacy of E966-0530-45418 in attenuating the progression of pulmonary fibrosis in a well-established animal model. We induced pulmonary fibrosis in C57BL/6 mice through intratracheal bleomycin administration, which has previously been described to lead to lung fibrosis and concurrent collagen accumulation after 21 days 38, 39. As depicted in **Figure 7A**, E966-0530-45418 was orally administered via gavage at either day 0 (preventative) or day 7 (therapeutic) post-bleomycin exposure, and at day 21, the extent of lung fibrosis in the mice was evaluated by WBP and microcomputed tomography; bronchoalveolar lavage fluid (BALF) was also collected for analysis, and body weight was monitored throughout.

The body weights of the sham and E966-0530-45418 group mice increased steadily (**Figure 7B**), which might indicate that E966-0530-45418 was not toxic to the mice. In contrast, the bleomycin-treated mice gradually became emaciated, and on day 21, the weights of the bleomycin group mice were significantly lower than those of the sham group mice. However, this weight loss was ameliorated by E966-0530-45418 in both the preventative and therapeutic models, and the preventive effect of the compound was better than that of pirfenidone administration at day 0 (**Figure 7B & Supplementary Figure S9**).

The pulmonary function results revealed impaired pulmonary function in the mice 21 days after bleomycin treatment, with significant peak-to-trough differences, higher Penh values, peak expiratory flow, and minute ventilation, indicating more substantial airway obstruction disease severity (**Figure 7C-E & Supplementary Figure S10**).

The mice in the E966-0530-45418 group showed a smoother respiratory waveform, a significantly reduced Penh value, peak expiratory flow and minute ventilation, indicating a significant improvement in lung function; this effect was superior to that of pirfenidone (**Figure 7C-E & Supplementary Figure S10**). Consistent with these observations, micro-CT imaging revealed marked lung parenchymal destruction and volume reduction in the mouse lungs following bleomycin treatment, whereas both preventative and therapeutic E966-0530-45418 administration preserved the lung structure and mitigated the volume reduction induced by bleomycin treatment (**Figure 7F, G**). Flow cytometric analysis with double staining for CD206 and TGFβ1 in the BALF to identify M2 alveolar macrophages revealed that bleomycin treatment significantly increased the number of macrophages with the M2 phenotype and that this effect was impeded by E966-0530-45418 administration (**Figure 7H, I**). Collectively, E966-0530-45418 ameliorated lung function decline, alleviated lung parenchyma and attenuated the number of M2 macrophage phenotypes of BALF in bleomycin-induced PF mice.

### E966-0530-45418 inhibited fibrotic protein expression in bleomycin-induced PF mice by inhibiting CDK8 activity

**Figure 8:**
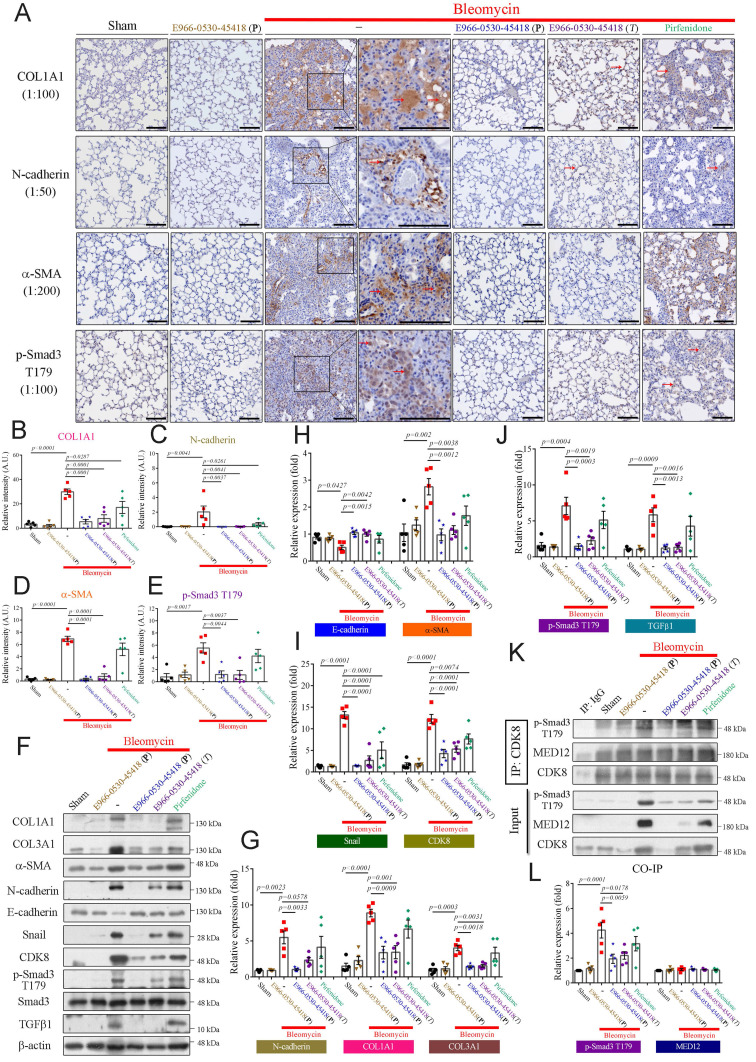
** E966-0530-45418 significantly mitigated bleomycin-induced EMT and myofibroblast differentiation in mouse lung tissue.** (**A**-**J**) IHC staining (200× magnification) of lung paraffin sections (Scale bar: 100 μm) (A-E) and western blot analysis of lung tissues (F-J) from the indicated groups of mice on day 22 with the indicated antibodies were used to evaluate specific protein expression (n = 5 independent animals per group). (**K**,** L**) Lung tissue proteins were immunoprecipitated with anti-CDK8 antibodies and subjected to immunoblotting to investigate the interaction between CDK8 and p-Smad3 T179 and MED12 (n = 5 independent animals per group). The results are shown as the mean ± SEM. *P* values were determined using one-way ANOVA followed by Tukey's post hoc test (B-E, G-J, and L).

We further performed immunohistochemical (IHC) staining to assess whether E966-0530-45418 obstructed EMT or myofibroblast differentiation after bleomycin treatment. IHC analysis demonstrated that the levels of the ECM protein COL1A1, the EMT protein N-cadherin, the myofibroblast marker α-SMA, and the active transcription factor p-Smad3 T179 were markedly increased in the bleomycin treatment group (**Figure 8A-E**). The lung tissue architecture was more consolidated in the bleomycin-treated group than in the sham group. In contrast, in the E966-0530-45418 administration group, the expression of these proteins was significantly attenuated, and lung consolidation was ameliorated; a similar but less pronounced effect was observed in the pirfenidone administration group (**Figure 8A-E**). Moreover, immunoblot analysis of lung tissue revealed that compound administration not only decreased the expression of COL1A1, COL3A1, α-SMA, N-cadherin, and p-Smad3 T179 but also significantly reduced the levels of another EMT protein, snail, and the profibrotic factor TGFβ1 and rescued the levels of the epithelial cell marker E-cadherin (**Figure 8F-J**). In addition, administration of E966-0530-45418 markedly decreased the bleomycin-induced collagen deposition in the lungs, as observed through picrosirius red staining analysis and hydroxyproline content measurement (**Supplementary Figure S8**).

Furthermore, we conducted immunoprecipitation assays to investigate whether E966-0530-45418 inhibited CDK8 cooperation with the Mediator complex to regulate the fibrotic process. Significant interactions between CDK8 and p-Smad3 T179 were observed in the bleomycin treatment group compared to the sham group, and treatment with E966-0530-45418 markedly blocked this interaction (**Figure 8K, L**). However, there were no changes in the interaction between CDK8 and MED12 in response to bleomycin, E966-0530-45418, or pirfenidone (**Figure 8K, L**). This finding of IP assays suggests it is an underlying mechanism for the inhibited activity of CDK8 by E966-0530-45418 to mitigate the fibrosis process *in vivo*. Together with the findings above, these results indicate that E966-0530-45418 ameliorates pulmonary fibrosis in the bleomycin-induced mouse model.

## Discussion

PF is an incurable lung condition marked by fibrosis that causes a gradual decline in pulmonary function and is difficult to treat with current medicines; therefore, we attempt to search for a new strategy for PF therapy. In this study, we demonstrated the effectiveness of E966-0530-45418 in attenuating EMT, cell migration, myofibroblast differentiation, and COL1A1, TGFβ1 and CTGF production in various cells, including A549 cells, WI-38 cells, and human primary AECs. Mechanistic investigations demonstrate that E966-0530-45418, through CDK8 inhibition, decreased the phosphorylation of Smad3 at T179 and RNA polymerase II at S2/5 to reduce the transcription of EMT and fibrosis association proteins. In addition, E966-0530-45418 also lowered the levels of p-STAT3 S727 to descend the number of M2 macrophages phenotype. Importantly, it was observed that E966-0530-45418 ameliorated lung dysfunction and lung parenchymal destruction in bleomycin-induced PF mice. These data suggest the therapeutic potential of E966-0530-45418 in patients with PF.

Fibrotic molecules are produced through the active TGFβ1/Smad signaling, inducing transcription that can be regulated by CDK8. In the initiation step of transcription, the Mediator kinase module containing CDK8 interacts with the core Mediator complex, which assembles with transcription factors and RNA polymerase II into the preinitiation complex, constructs a link with promoters, and anchors the preinitiation complex to gene-specific upstream enhancers, and all of these steps are important for transcriptional regulation 40, 41. CDK8 binds cyclin C to acquire basal activity and further interacts with MED12 to achieve the full scope of its activity; hence, the combination of CDK8, cyclin C, MED12, and MED13 is referred to as the Mediator kinase module 14. Smad3 is phosphorylated at the T179 site through CDK8 to maintain transcriptional activity 33, and CDK8 phosphorylates RNA polymerase II at the S2/5 site to enhance the execution of transcription 15, which increases target gene expression. In this study, E966-0530-45418 treatment reduced the expression of p-Smad3 T179 and p-RNA polymerase II S2/5 (**Figure 4A, B; Supplementary Figure S4A, B & S5A, B**) and decreased Smad3 binding to the promoters of EMT, COL1A1, and fibrotic factor genes (**Figure 4I-K**); furthermore, it downregulated the transcriptional activity of EMT genes (**Figure 4M**). The data from the bleomycin-induced pulmonary fibrosis animal model also supported the *in vitro* results, demonstrating that E966-0530-45418 administration significantly decreased the phosphorylation of Smad3 at T179, further reducing fibrosis-associated protein expression (**Figure 8**).

Interestingly, we observed an increase in CDK8 and MED12 expression with bleomycin treatment* in vivo* (**Figure 8K**), as well as increased CDK8 expression in IPF patients (**Figure 1D, E**), whereas in cell experiments, treatment with TGFβ did not increase the expression of CDK8 or MED12 (**Figure 4E**). Notably, analysis of the GSE191279 dataset indicated that prolonged exposure of alveolar epithelial cells (AECs) to TGFβ (6 days) resulted in elevated CDK8 mRNA expression, which may provide a mechanistic explanation for the increased CDK8 expression observed in lung fibrosis (**Supplementary Figure S11**). This finding may suggest that a longer duration of bleomycin-induced lung fibrosis in animal models may induce a more comprehensive fibrotic process. The increase in CDK8 expression may be related to the regulation of miRNA-770, as previous studies have shown an inverse correlation between miRNA-770 expression and CDK8 expression 42, and miRNA-770 is inhibited by TGFβ1 in lung fibrosis 43. On the other hand, Priya et al. reported that gain-of-function mutations in MED12 lead to fibrosis and ECM deposition in leiomyomas 44, and Ayman et al. reported that silencing MED12 reduces the expression of fibrosis-associated proteins 45; these studies suggest that increased activity or expression of MED12 may lead to organ fibrosis, similar to our observations in animal models. The ability of E966-0530-45418 to reduce the increases in CDK8 and MED12 induced by bleomycin *in vivo* requires further investigation to fully understand the molecular basis of E966-0530-45418 function.

The role of the inflammatory response in the progression of PF has been a subject of debate. While chronic inflammation was previously believed to be the primary cause of IPF initiation and exacerbation, more recent work has revealed that aberrant wound healing is also a significant factor in initiating and aggravating IPF 46. Clinical trials have shown that IPF patients treated with a combination regimen comprising the anti-inflammatory corticosteroid prednisone, the immunosuppressant azathioprine, and the mucolytic N-acetylcysteine face increased risks of mortality and hospitalization 47. Additionally, another corticosteroid, dexamethasone, was ineffective in mitigating the pro-fibrotic effects of TGFβ1 48. These findings suggest that targeting pro-inflammatory responses may not be essential for PF therapy. However, other studies have highlighted the critical role of lung M2 macrophages in PF progression, which is likely due to their involvement in TGFβ1 production and consistent with their polarization via STAT3 phosphorylation 8, 9. Moreover, dexamethasone and azathioprine enhance macrophage polarization toward the M2 subtype, possibly explaining their inefficacy in IPF treatment 49, 50. Therefore, this study also focused on anti-inflammatory responses, mainly targeting M2 macrophages associated with PF. We demonstrated a significant reduction in the M2 macrophage population and in TGFβ1 production in both *in vitro* (**Figure 5D-H**) and *in vivo* models (**Figure 7H, I**) following E966-0530-45418 treatment, highlighting another beneficial aspect of this compound in PF therapy.

PF is often associated with an imbalance in protein expression; therefore, the primary purpose of treating this disease is to prevent or alleviate the pathophysiological mechanisms resulting from this imbalance. CDK8 may be a promising candidate target because it plays an important role in transcriptional regulation. Studies have shown that CDK8 positively regulates gene-specific transcription and has different roles in specific environments 20; as shown in this study, CDK8 collaboration with different transcription factors likely regulates the expression of various genes. Furthermore, the other benefit of CDK8 as a target is that its activity is unnecessary for general transcription and does not affect normal cell growth 13. Deleting CDK8 does not affect the homeostasis of the adult mouse intestine, characterized by high levels of cell proliferation and differentiation 24. In this study, E966-0530-45418 did not reduce the viability of A549, WI-38, and THP-1 cells or the human primary AECs, nor did it affect the cell cycle distribution (**Figure 2C-G**). Moreover, E966-0530-45418 administration did not lead to weight loss in mice (**Figure 7B**), indicating its safety in these mammals. However, long-term monitoring of potential adverse reactions may still be necessary to ensure safe therapeutic application. Moreover, E966-0530-45418 has not yet entered clinical trials, and its safety and ability to ameliorate PF lung damage in humans need to be evaluated.

E966-0530-45418 is a dual inhibitor targeting CDK8 and CDK19. While this study primarily discusses the inhibitory effects of E966-0530-45418 on CDK8 to mitigate PF, it provides limited insight into the impact on CDK19 activity or safety concerns. Although a study has shown that functional complementation between CDK8 and CDK19, as well as double CDK8/CDK19 knockout, does not significantly affect intestinal cell differentiation 24, further *in vivo* studies specifically targeting CDK19 inhibition and longer-term monitoring could enhance our understanding of its role in organismal effects.

Overall, this study revealed E966-0530-45418, a CDK8 inhibitor, for the first time to target CDK8 in an attempt to treat pulmonary fibrosis. The mechanism involves inhibition of the phosphorylation of Smad3 at T179, inhibiting the TGFβ1/Smad signaling pathway and subsequently impeding the phosphorylation of RNA polymerase II at S2/5. Ultimately, this reduction in phosphorylation leads to decreased EMT and FMT in AECs and lung fibroblasts, thereby blocking their differentiation into myofibroblasts and reducing ECM deposition. Additionally, E966-0530-45418 decreases the levels of p-STAT3 S727 in the IL6/STAT3 signaling pathway, resulting in a decrease in M2 macrophage polarization. Furthermore, our experiment showed that the efficacy of E966-0530-45418 is superior to that of pirfenidone in both *in vitro* and *in vivo*. Together, these findings indicate that E966-0530-45418 may be a promising drug for mitigating the progression of PF.

## Supplementary Material

Supplementary methods, figures and tables.

## Abbreviations

α-SMA: alpha smooth muscle actin
AECs: Alveolar epithelial cells
BALF: Bronchoalveolar lavage fluids
CCNC: Cyclin C
CDK8: Cyclin-dependent kinase 8
ChIP: Chromatin immunoprecipitation
COL1A1: Collagen type I alpha 1 chain
COL3A1: Collagen type III alpha 1 chain
CTGF: Connective tissue growth factor
ECM: Extracellular matrix
EMT: Epithelial-mesenchymal transition
FMT: Fibroblast-to-myofibroblast transition
GEO: Gene Expression Omnibus
GSEA: Gene set enrichment analysis
IHC: Immunohistochemistry
IL6: Interleukin 6
IP: immunoprecipitation
IPF: Idiopathic pulmonary fibrosis
IT: Intratracheal
μCT: Microcomputed tomography
MED12: Mediator complex subunit 12
PEF: Peak expiratory rate
Penh: Enhanced pause
PF: Pulmonary fibrosis
PMA: Phorbol 12-myristate 13-acetate
POLR2A: RNA polymerase II subunit A
PPP1R15A: Protein Phosphatase 1 Regulatory Subunit 15A
PRKCZ: Protein Kinase C Zeta
qPCR: Quantitative polymerase chain reaction
STAT3: Signal transducer and activator of transcription 3
TGFβ1: Transforming growth factor beta 1
TRIM33: tripartite motif containing 33
WBP: Whole body plethysmography
