# Supplementary material for: The Cyclin-Dependent Kinase 8 Inhibitor E966-0530-45418 Attenuates Pulmonary Fibrosis In Vitro and In Vivo
Source: Int J Biol Sci. 2025 Jan 1;21(2):685–707. doi: 10.7150/ijbs.105826 (PMC11705631; doi:10.7150/ijbs.105826)
Supplement: Supplementary file 1 — Supplementary methods, figures and tables. [file ijbsv21p0685s1.pdf]

Supplementary materials

The Cyclin-Dependent Kinase 8 Inhibitor E966–0530–45418 Attenuates Pulmonary Fibrosis *in Vitro* and *in Vivo*

Ching-Hsuan Chou[1], Wei-Jan Huang[2,3], Kai-Cheng Hsu[4,5], Jui-Yi Hsu[4,5], Tony Eight Lin[4,5], Chia-Ron Yang[1,*]

[1] School of Pharmacy, College of Medicine, National Taiwan University, Taipei, Taiwan

[2] School of Pharmacy, Taipei Medical University, Taipei, Taiwan

[3] Graduate Institute of Pharmacognosy, College of Pharmacy, Taipei Medical University, Taipei, Taiwan

[4] Graduate Institute of Cancer Biology and Drug Discovery, College of Medical Science and Technology, Taipei Medical University, Taipei, Taiwan

[5] Ph.D. Program for Cancer Molecular Biology and Drug Discovery, College of Medical Science and Technology, Taipei Medical University, Taipei, Taiwan

* Correspondence author: Chia-Ron Yang

School of Pharmacy, College of Medicine, National Taiwan University, Taipei, Taiwan

Tel: 886-2-33668758; Fax: 886-2-23919098; E-mail address: cryang@ntu.edu.tw

Supplementary methods

Histology

Mouse left lungs were fixed with 4% formaldehyde, embedded in paraffin, and sectioned in 5 μm thickness for picrosirius red staining. The fibrotic area was measured using ImageJ software.

Hydroxyproline assay

Hydroxyproline levels in lung tissues were measured using the Hydroxyproline Colorimetric Assay kit (BioVision, Milpitas, CA, USA). Appropriate frozen right lung tissues homogenized in 10 μL ddH_2O/mg tissue followed by equivalent hydrochloric acid (HCl, 12 N) for hydrolysis at 120 °C for 3 h. Then, 10 μL of individual samples were taken to quantify the absorbance at 560 nm. Hydroxyproline content was presented in microgram per milligram tissue.

Supplementary figure 1

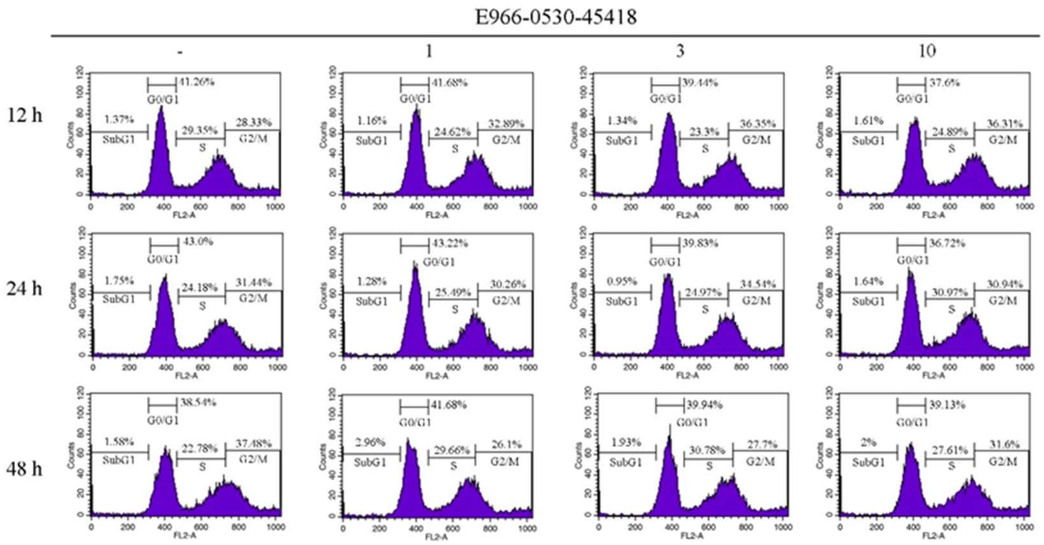

Figure S1. E966–0530–45418 does not affect cell cycle progression, related to Figure 2. Flow cytometric analysis of PI staining was used to evaluate the cell cycle distribution of human primary AECs treated with or without different concentrations (1, 3, or 10 μM) of E966–0530–45418 for 12, 24, or 48 h. Actual flow cytometry results correspond to Figure 2G.

Supplementary figure 2

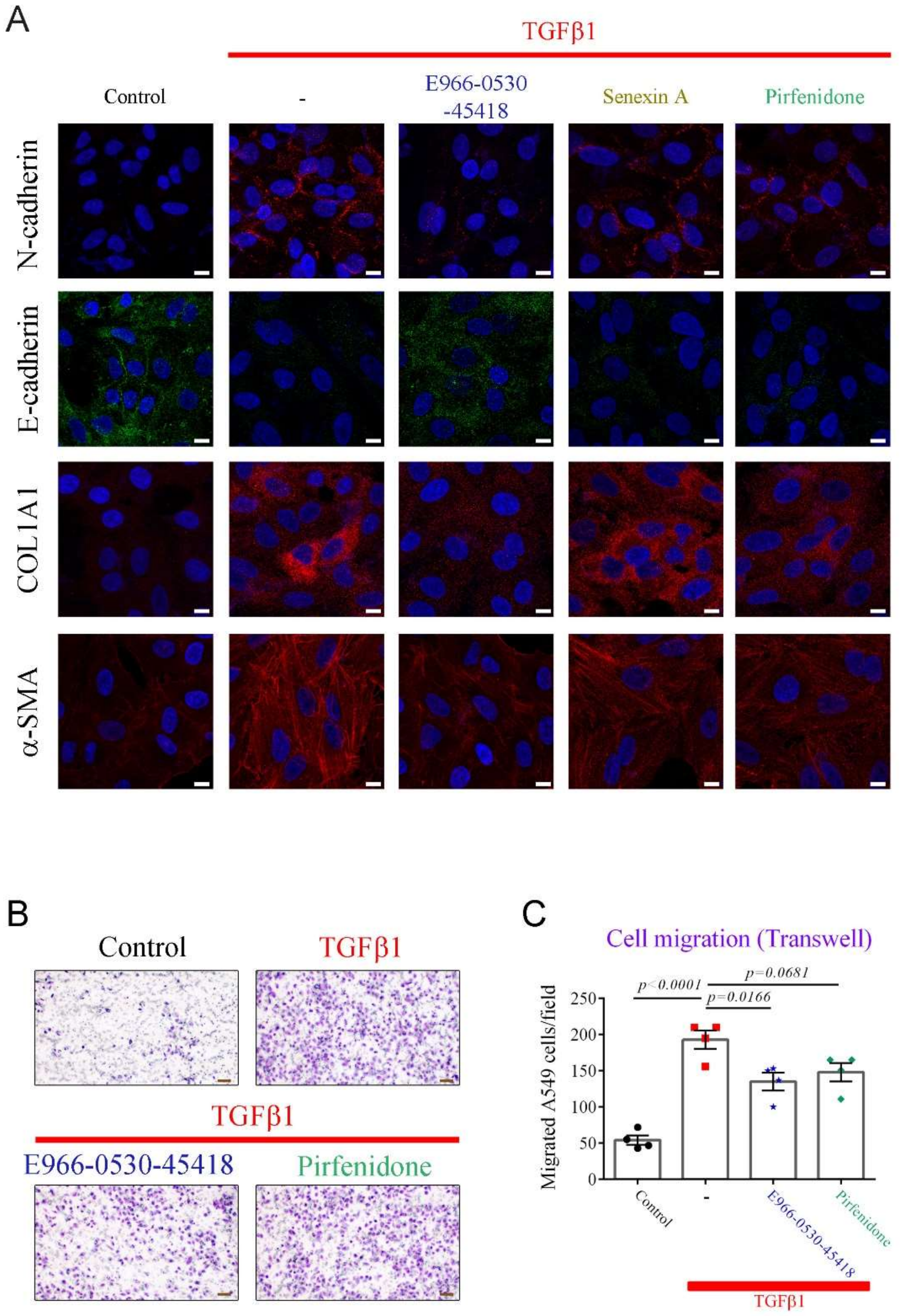

Figure S2. E966–0530–45418 reduced the increases of EMT, fibrosis-related

markers expression, and cell migration induced by TGFβ1 treatment in A549 cells. It was related to Figure 3. (**A**) A549 cells were exposed to E966–0530–45418 (5 μM), senexin A (5 μM), pirfenidone (1 mM), or no inhibitor in the presence of TGFβ1 (10 ng/mL) for 24 h. Immunofluorescence images of A549 cells were acquired with a Zeiss LSM880 confocal microscope at 400 × magnification (Scale bar: 10 μm). (**B**, **C**) A549 cells were seeded in Transwell plates and treated with or without E966-0530-45418 (5 μM) pirfenidone (1 mM) or no inhibitor in the presence of TGFβ1 (10 ng/mL) for 18 h. The migrated cells were stained with crystal violet and imaged under a microscope (40× magnification) (Scale bar: 100 μm) (B). The mean number of migrated cells was quantified using ImageJ software (C) (n = 4 independent samples per group). The results are shown as the mean ± SEM. *P* values were determined using one-way ANOVA followed by Tukey's post hoc test (C).

Supplementary figure 3

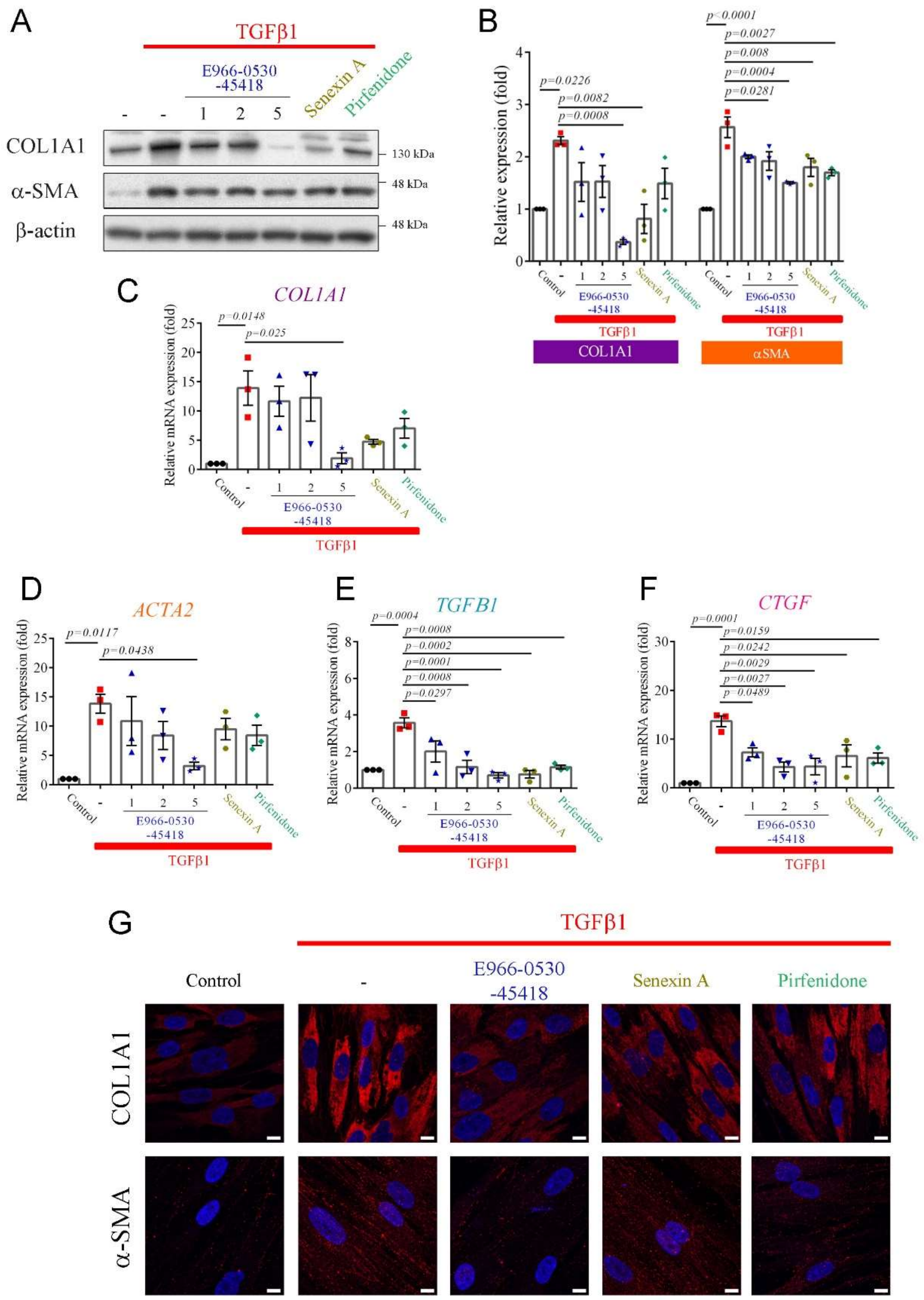

Figure S3. The levels of TGFβ1-induced FMT proteins and fibrotic markers were

markedly reduced by E966–0530–45418 treatment in WI-38 cells. It was related to Figure 3. (**A**–**G**) WI-38 cells were exposed to the indicated concentrations of E966–0530–45418 (μM, panel G is 5 μM), senexin A (5 μM), pirfenidone (1 mM), or no inhibitor in the presence of TGFβ1 (10 ng/mL) for 24 h. The protein levels of COL1A1 and α-SMA were determined by western blotting in WI-38 cells (A and B). The mRNA levels of COL1A1, α-SMA, TGFβ1, and CTGF were analyzed by RT–qPCR in WI-38 cells (C–F) (A–F, n = 3 independent samples per group). The results are shown as the mean ± SEM. *P* values were determined using one-way ANOVA followed by Tukey's post hoc test (B–F). Immunofluorescence images of WI-38 cells were acquired with a Zeiss LSM880 confocal microscope at 400 × magnification (Scale bar: 10 μm) (G).

Supplementary figure 4

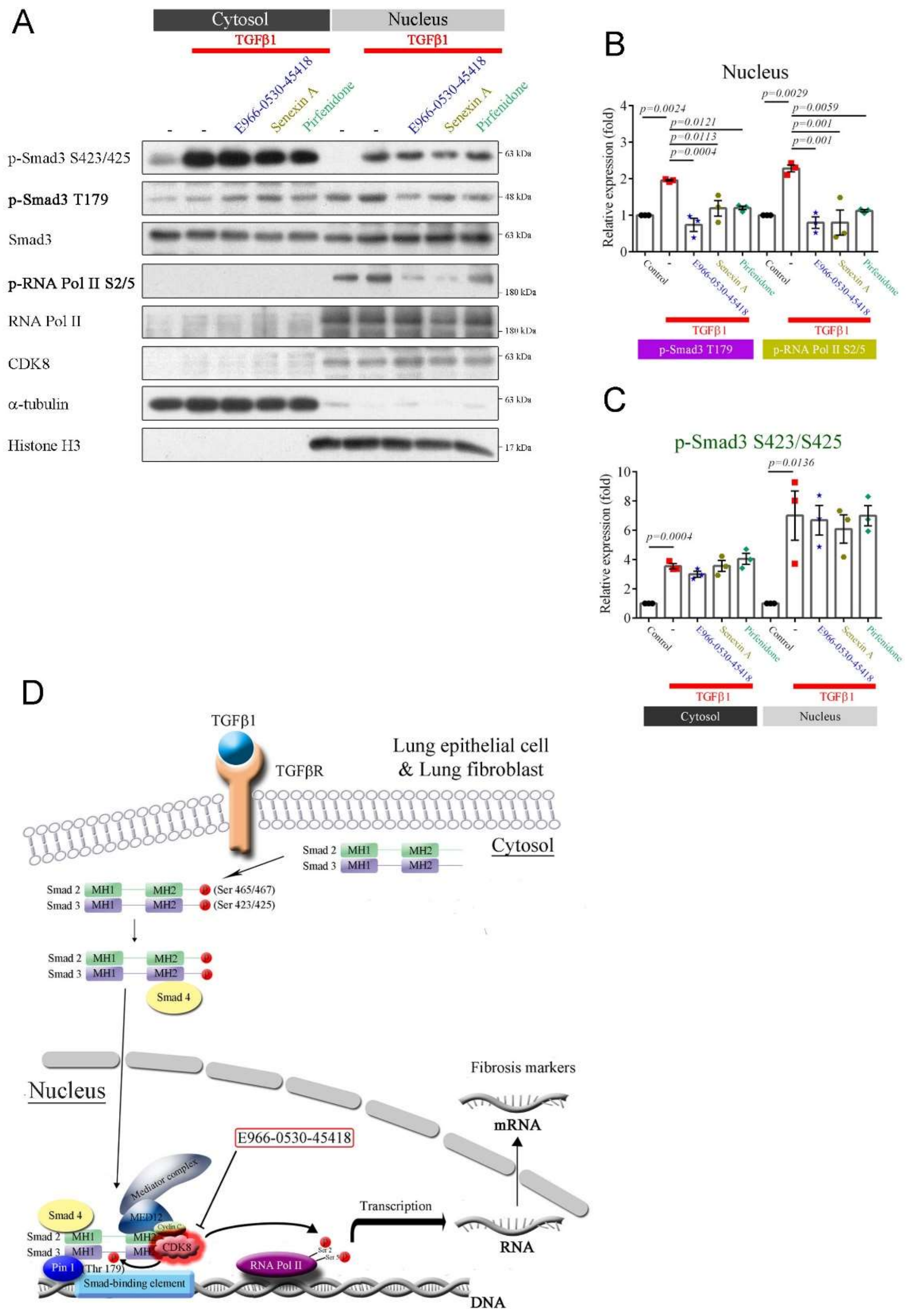

Figure S4. E966–0530–45418 significantly attenuated TGFβ1/Smad3/RNA

polymerase II signal transduction. It was related to Figure 4. (**A**–**C**) WI-38 cells were treated with E966–0530–45418 (5 μM), senexin A (5 μM), pirfenidone (1 mM), or no inhibitor in the presence of TGFβ1 (10 ng/mL) for 3 h and then subjected to nuclear-cytosolic fractionation. The protein levels in the cytosol and nucleus were detected by western blotting and quantified (n = 3 independent samples per group). The results are shown as the mean ± SEM. *P* values were determined using one-way ANOVA followed by Tukey's post hoc test (B and C). (**D**) Schematic diagram of the proposed mechanism: E966–0530–45418 inhibits the CDK8-mediated phosphorylation of both Smad3 at T179 and RNA Pol II at S2/5, which might attenuate the transcription of specific genes involved in the active TGFβ1/Smad3 signaling pathway.

Supplementary figure 5

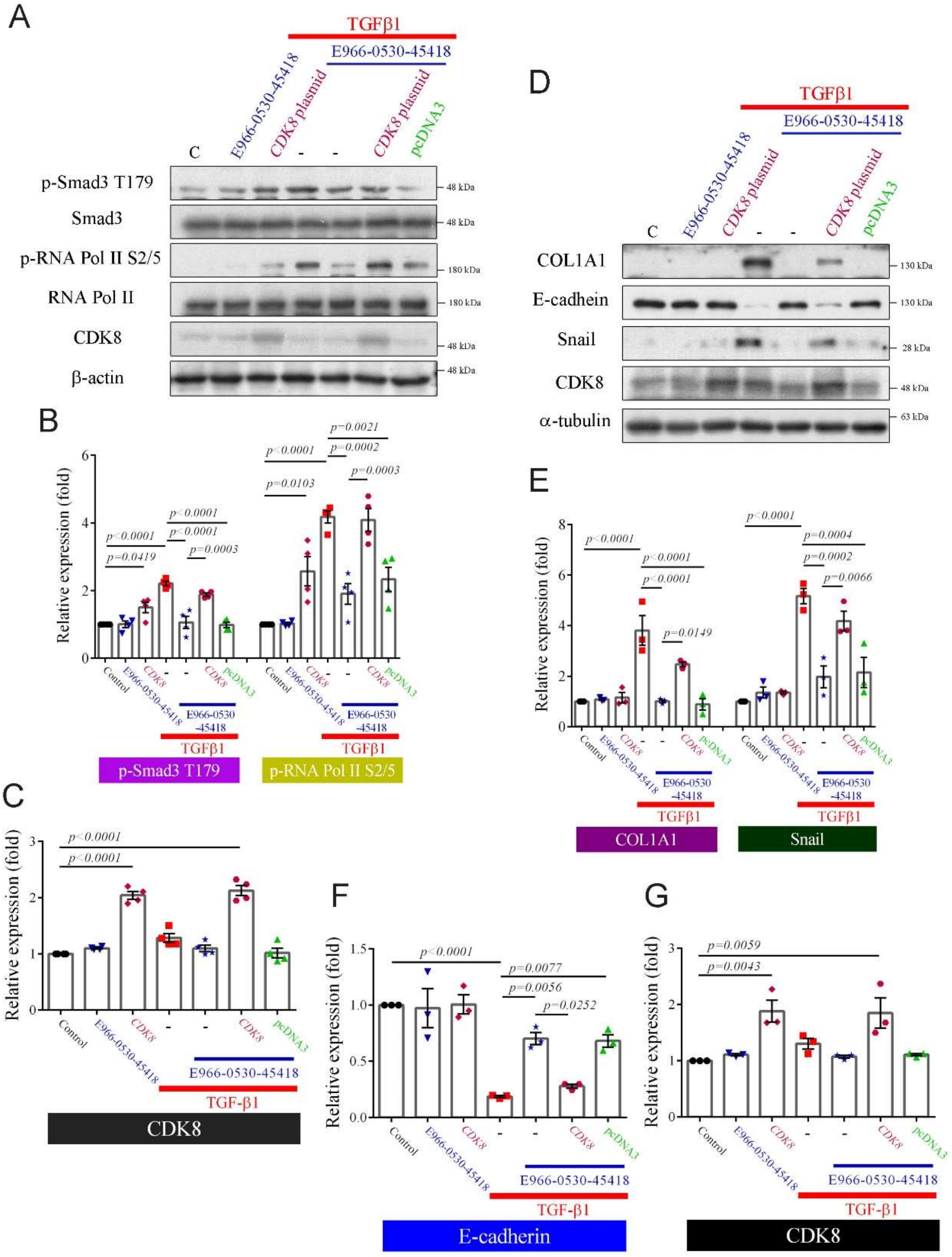

Figure S5. CDK8 overexpression reversed the suppressive effects of E966–0530–

45418 on EMT-related genes and myofibroblast formation in TGFβ1-induced A549 cells. It was related to Figure 4. (**A**–**G**) A549 cells were transfected with the pcDNA3 *CDK8-HA* plasmid (1 μg) for 24 h, treated with E966–0530–45418 (5 μM) for 1 h, and then incubated with TGFβ1 (10 ng/mL) for an additional 3 h (A–C) (n = 4 independent samples per group) or 24 h (D–G) (n = 3 independent samples per group), and then whole-cell lysates were subjected to western blotting with the indicated antibodies. The results are shown as the mean ± SEM. *P* values were determined using one-way ANOVA followed by Tukey's post hoc test (B–C and E–G).

Supplementary figure 6

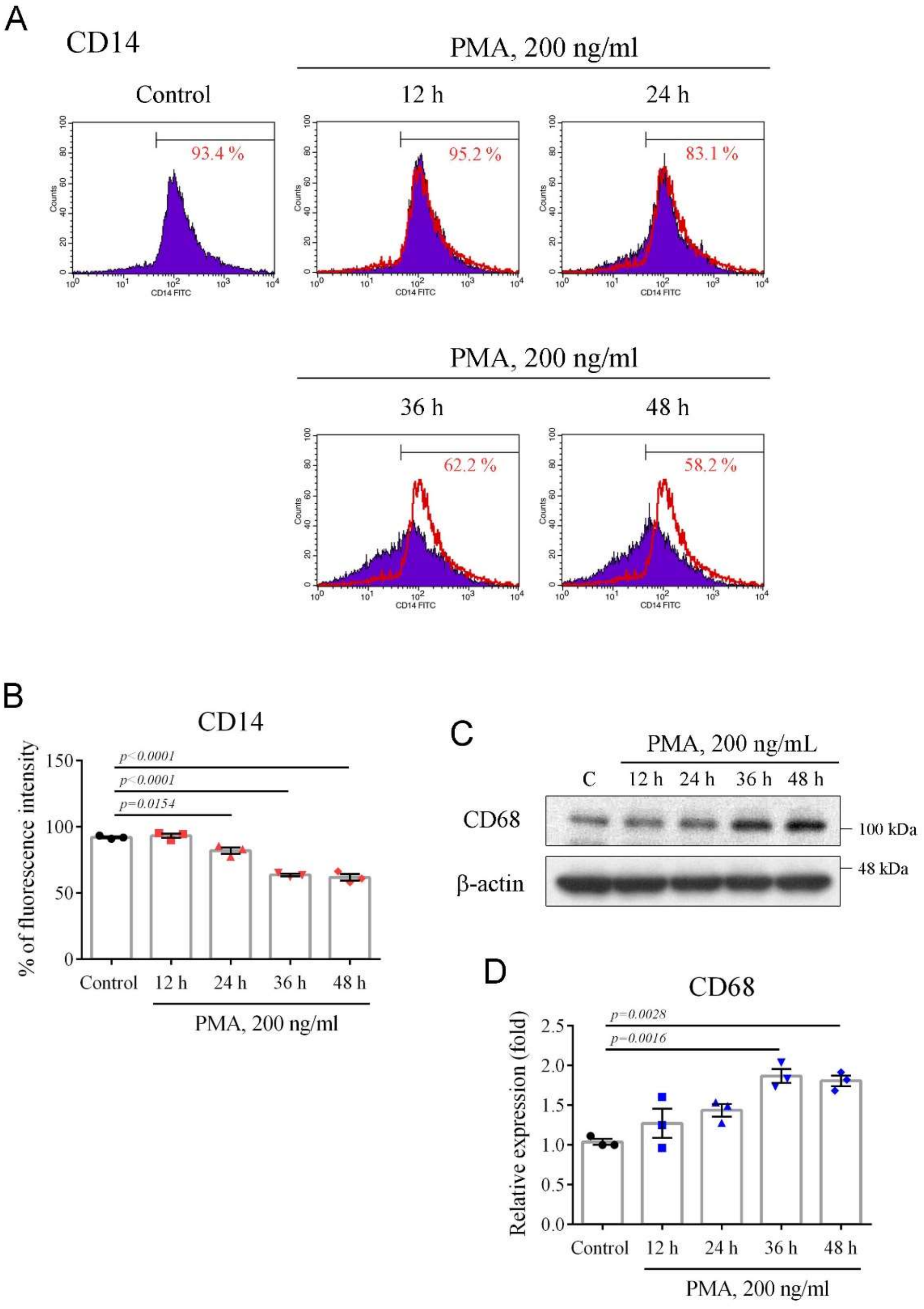

Figure S6. CD14 proteins were markedly reduced and CD68 proteins were

significantly increased in response to PMA after 36 h in THP-1 cells. It was related to Figure 5. (**A**–**D**) THP-1 cells were exposed to PMA (200 ng/mL) for the indicated duration. (A, B) Flow cytometric analysis was used to test the expression of CD14 in THP-1 cells by FITC anti-human CD14 antibody (Biolegend, 301804, 5 µl per million cells in 100 µl volume) staining. (C, D) Western blot analysis was used to evaluate the expression of CD68 in THP-1 cells by CD68 antibody (Abclonal, A6554). (A–D, n = 3 independent samples per group). The results are shown as the mean ± SEM. *P* values were determined using one-way ANOVA followed by Tukey's post hoc test (B and D).

Supplementary figure 7

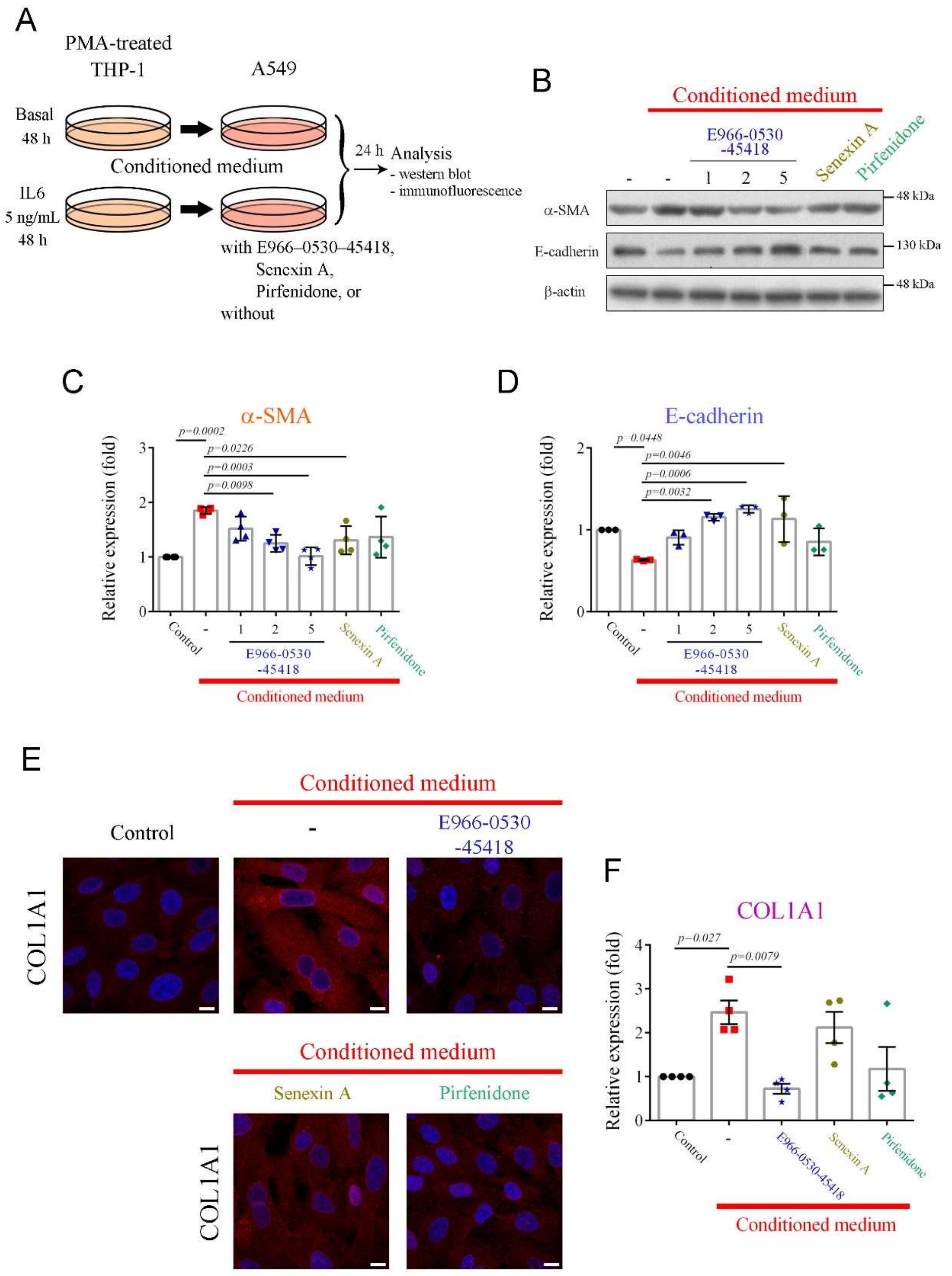

Figure S7. E966–0530–45418 markedly obstructed the fibrotic and EMT processes

in A549 cells caused by the conditioned medium from the IL6-treated PMA-induced THP-1 cells, related to Figure 5. (**A**) Illustration of the experimental procedure. After IL6 treating PMA-induced THP-1 cells for 48 h, the conditioned medium from which and the culture medium for A549 were mixed in equal proportions to incubate A549 cells for 24 h. (**B**–**F**) The protein levels of α-SMA and E-cadherin were determined by western blotting (B–D), and COL1A1 were evaluated by immunofluorescence analysis (Scale bar: 10 μm) (E and F) in A549 cells (n = 4 independent samples per group except for E-cadherin that is n = 3). The results are shown as the mean ± SEM. *P* values were determined using one-way ANOVA followed by Tukey's post hoc test (C, D, and F).

Supplementary figure 8

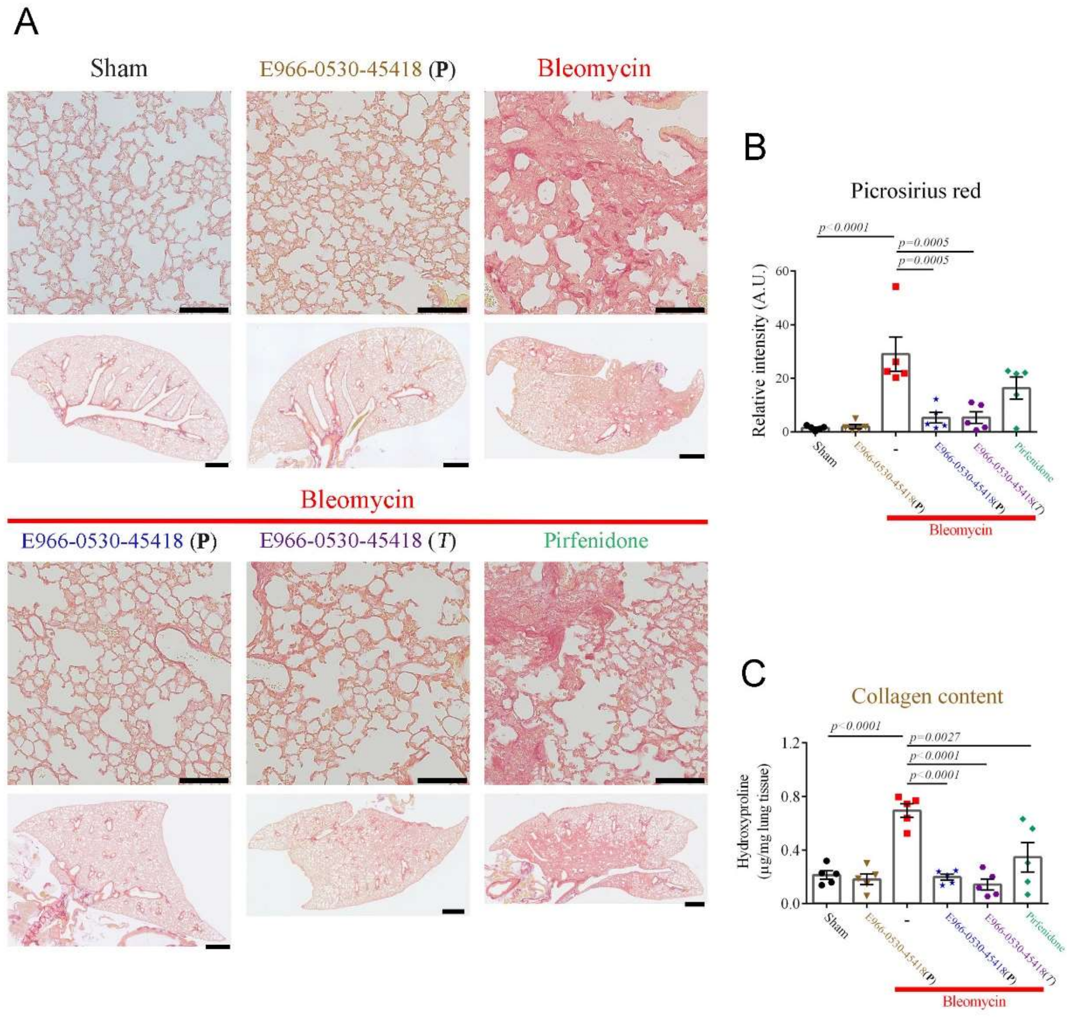

Figure S8. E966–0530–45418 significantly attenuated collagen deposition of the lung in bleomycin-provoked PF mice. It was related to Figure 8. (**A**–**C**) Collagen deposition in the lung was evaluated by picrosirius red staining of lung sections (Scale bar; upper: 100 μm, lower: 1 mm) (A and B) and hydroxyproline levels of lung tissues (C) from the indicated groups of mice on day 22 (n = 5 independent animals per group). The results are shown as the mean ± SEM. *P* values were determined using one-way

ANOVA followed by Tukey's post hoc test (B and C).

Supplementary figure 9

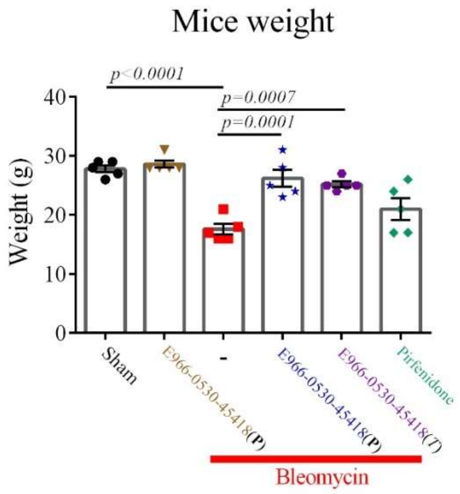

Figure S9. E966–0530–45418 significantly alleviated weight loss in bleomycin-induced PF mice. This result is related to Figure 7. Body weight was evaluated on day 20 after bleomycin treatment. The results are shown as the mean ± SEM (n = 5 independent animals per group). *P* values were determined using one-way ANOVA followed by Tukey's post hoc test.

Supplementary figure 10

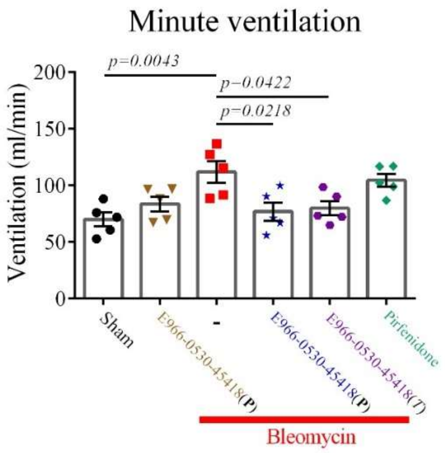

Figure S10. E966–0530–45418 significantly mitigated the elevated minute ventilation observed in bleomycin-induced PF mice. This finding is associated with Figure 7. Barometric plethysmography was performed on day 21 to assess pulmonary respiratory function across different groups of mice. Minute ventilation values were calculated as an in vivo indicator of lung fibrosis severity. Results are presented as the mean ± SEM (n = 5 independent animals per group). *P* values were determined using one-way ANOVA followed by Tukey's post hoc test.

Supplementary figure 11

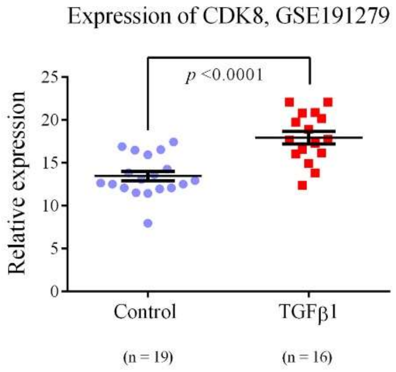

Figure S11. CDK8 expression was elevated following 6 days of TGFβ1 treatment in alveolar epithelial cells. *CDK8* mRNA levels were compared between TGFβ1-treated and untreated alveolar epithelial cells isolated from human lung tissue, as derived from the GSE191279 dataset. Statistical significance was assessed using a two-tailed unpaired Student's t-test, with results presented as the mean ± SEM.

Supplementary figure 12

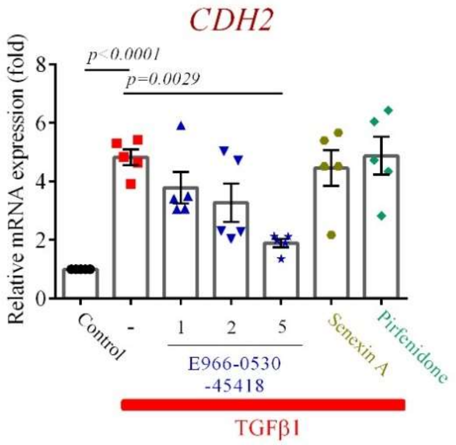

Figure S12. E966–0530–45418 significantly reduced the TGFβ1-induced increase in *CDH2* mRNA levels in a concentration-dependent manner in A549 cells. A549 cells were exposed to the indicated concentrations of E966–0530–45418 (1, 2, 5 μM), senexin A (5 μM), pirfenidone (1 mM), or no inhibitor, in the presence of TGFβ1 (10 ng/mL) for 24 h. The mRNA levels of *CDH2* were analyzed by qPCR in A549 cells (n = 5 independent samples per group). Results are presented as the mean ± SEM. P values were determined using one-way ANOVA followed by Tukey's post hoc test.

Supplementary figure 13

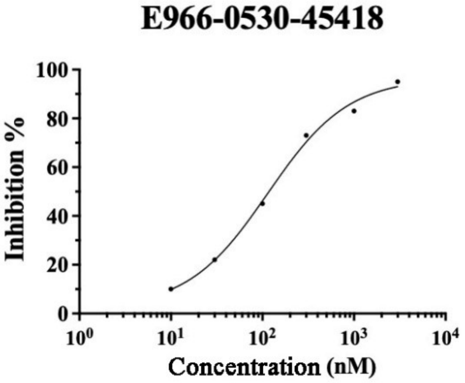

Figure S13. Concentration-dependent inhibition of CDK8 by E966–0530–45418. The inhibition of CDK8 activity by E966–0530–45418 was evaluated using an in vitro kinase assay, with an IC_{50} value of 129 nM. The graph represents the percentage inhibition plotted against the compound concentration (nM) on a logarithmic scale. Nonlinear regression (Inhibitor vs. Response – Variable Slope) was performed using GraphPad Prism software version 6.01.

Supplementary Table S1. Antibodies and plasmids were used in this study.

Materials	Venders	Catalog number & Identifier
Antibodies		
N-Cadherin (D4R1H) Rabbit mAb	Cell Signaling Technology	Cat# 13116; RRID:AB_2687616
Snail (C15D3) Rabbit mAb	Cell Signaling Technology	Cat# 3879; RRID:AB_2255011
SMAD3 (C67H9) Rabbit mAb	Cell Signaling Technology	Cat# 9523; RRID:AB_2193182
Phospho-Rpb1 CTD (Ser2/Ser5) (D1G3K) Rabbit mAb	Cell Signaling Technology	Cat# 13546; RRID:AB_2798253
MED12 (D9K5J) Rabbit mAb	Cell Signaling Technology	Cat# 14360; RRID:AB_2798461
Histone H3 Antibody	Cell Signaling Technology	Cat# 9715; RRID:AB_331563
Rabbit Anti-Stat-3 Monoclonal Antibody	Abcam	Cat# 2281-1; RRID:AB_1267343
Rabbit Anti-Stat-3, phospho (Ser727) Monoclonal Antibody	Abcam	Cat# 1121-1; RRID:AB_344887
Rabbit Anti-Stat-3, phospho (Tyr705) Monoclonal Antibody	Abcam	Cat# 2236-1; RRID:AB_1267344
Anti-alpha smooth muscle Actin antibody	Abcam	Cat# ab5694; RRID:AB_2223021
E-Cadherin Rabbit mAb	ABclonal	Cat# A20798
Collagen I/COL1A1 Rabbit pAb	ABclonal	Cat# A16891; RRID:AB_2768989
Phospho-Smad3-T179 Rabbit pAb	ABclonal	Cat# AP0554; RRID:AB_2771545
Phospho-Smad3-S423/S425 Rabbit mAb	ABclonal	Cat# AP0727; RRID:AB_2863813
POLR2A Rabbit pAb	ABclonal	Cat# A11181; RRID:AB_2758448
Cyclin C Rabbit pAb	ABclonal	Cat# A6545;

		RRID:AB_2767138
Arginase 1 (ARG1) Rabbit mAb	ABclonal	Cat# A4923; RRID:AB_2863390
TGF beta 1 Rabbit pAb	ABclonal	Cat# A2124; RRID:AB_2764143
β-Actin Rabbit mAb (High Dilution)	ABclonal	Cat# AC026; RRID:AB_2768234
CDK8 antibody [C3], C-term	Genetex	Cat# GTX110495; RRID:AB_2036538
alpha Smooth Muscle Actin antibody	Genetex	Cat# GTX100034; RRID:AB_1240408
alpha Tubulin antibody	Genetex	Cat# GTX112141; RRID:AB_10722892
Cdk8 Antibody (D-9)	Santa Cruz Biotechnology	Cat# sc-13155; RRID:AB_627244
Pin1 Antibody (G-8)	Santa Cruz Biotechnology	Cat# sc-46660; RRID:AB_628132
FITC anti-human CD14 Antibody	BioLegend	Cat# 301804; RRID:AB_314186
FITC anti-human CD206 (MMR) Antibody	BioLegend	Cat# 321104; RRID:AB_571905
PE anti-mouse CD206 (MMR) Antibody	BioLegend	Cat# 141706; RRID:AB_10895754
FITC anti-mouse LAP (TGF-β1) Antibody	BioLegend	Cat# 141414; RRID:AB_2721328
Anti-rabbit IgG, HRP-linked Antibody	Cell Signaling Technology	Cat #7074; RRID:AB_2099233
Anti-mouse IgG, HRP-linked Antibody	Cell Signaling Technology	Cat #7076; RRID:AB_330924
Goat Anti-Rabbit IgG antibody (DyLight594)	Genetex	Cat# GTX213110-05; RRID:AB_2887580
Goat Anti-Rabbit IgG antibody (DyLight488)	Genetex	Cat# GTX213110-04; RRID:AB_2887579
Plasmids		
pcDNA3 *CDK8 HA*	Addgene (*from* Matija Peterlin)	#14649 RRID: Addgene_14649
7TFP *CDH1* reporter	Addgene (*from* Bob Weinberg)	#91704 RRID: Addgene_91704

pGL3-*TGFB1* reporter	Addgene<br>(*from* Yuh-Shan Jou)	#101762<br>RRID: Addgene_101762

Supplementary Table S2. Primer sequences were used in this study.

A. qRT-PCR Primers

Gene Name	Species	Sequence
CDH1 (E-cadherin)	human	AAAGGCCCATTTCCTAAAAACCT
		TGCGTTCTCTATCCAGAGGCT
SNAI1 (Snail)	human	GAGGACAGTGGGAAAGGCTC
		TGGCTTCGGATGTGCATCTT
COL1A1	human	GAGGGCCAAGACGAAGACATC
		CAGATCACGTCATCGCACAAC
ACTA2 (α-SMA)	human	AAAAGACAGCTACGTGGGTGA
		GCCATGTTCTATCGGGTACTTC
TGFB1 (TGFβ1)	human	CTAATGGTGGAAACCCACAACG
		TATCGCCAGGAATTGTTGCTG
CCN2 (CTGF)	human	GCGTGTGCACCGCCAAAGAT
		CAGGGCTGGGCAGACGAACG
GAPDH	human	CCATCACCATCTTCCAGGAGCG
		AGAGATGATGACCCTTTTGGC

B. ChIP-qPCR Primers

Gene Name	Species	Sequence
CDH2 promoter (N-cadherin)	human	AGTACATCCTCAAGGGTGGG
		TCATTCTTTGGAGATGGGTA
SNAI1 promoter (Snail)	human	CAGGTGACCCGCCTCTTAAC
		AGGGTAGCTTCTGGTCCAGT
COL1A1 promoter	human	CATTCCCAGCTCCCCTCTCT
		AGTCTACGTGGCAGGCAAGG
ACTA2 promoter (α-SMA)	human	CAGCTGGTCATGGCTGTAAAATAAAG
		CTCATAAAGAAATATTTTGTGGGTACTG
TGFB1 promoter (TGFβ1)	human	GCAACTTCGACCGCTACGG
		CTGCGACCCCATACATTTACTG
CCN2 promoter (CTGF)	human	AGTGGTGCGAAGAGGATAGG
		CATTCCTCGCATTCCTCCCC
